# Non-coding RNAs in dermatophytosis: Key regulators of host-fungal interactions and pathogenesis-A review

**DOI:** 10.1080/21505594.2026.2712727

**Published:** 2026-08-09

**Authors:** Jun Wan, Xixi Zhu, Jiayu Zheng, Bishibei Li, Ruihuan Zhang, Fangfang Zang, Yiping Hu, Yinpengxi Chen, Ziyu Wang, Kui Yang, Chunxiang Zhang, Wudian Xiao

**Affiliations:** a School of Basic Medical Sciences, Basic Medicine Research Innovation Center for Cardiometabolic Diseases, Ministry of Education, Luzhou, China; b Department of Cardiology, The Affiliated Hospital of Southwest Medical University, Luzhou, China; c Key Laboratory of Medical Electrophysiology, Ministry of Education, Luzhou, China; d Institute of Cardiovascular Research, Nucleic Acid Medicine of Luzhou Key Laboratory, Model Animal and Human Disease Research of Luzhou Key Laboratory, Luzhou, China; e Laboratory Animal Center, School of Clinical Medicine, School of Public Health, Southwest Medical University, Luzhou, China; f College of Intelligent Manufacturing, College of Science and Technology Changchun, Changchun, China

**Keywords:** Dermatophytosis, non-coding RNAs, Host-pathogen interaction, fungal virulence, skin immunity

## Abstract

Dermatophytosis, a prevalent superficial fungal infection caused by fungi such as *Trichophyton rubrum*, *Trichophyton mentagrophytes*, and *Microsporum canis*, develops through the dynamic interaction between fungal virulence factors – including adhesion, secretion of keratinolytic proteases, and hyphal invasion – and the host’s multifaceted immune response, which involves antimicrobial peptides, pattern recognition receptors, and cellular immunity. Recent evidence identifies non-coding RNAs (ncRNAs), including miRNAs, lncRNAs, circRNAs, and tRFs, as key regulators of this host-fungal crosstalk. This review synthesizes current advances, showing that dermatophyte infection alters host ncRNA expression while fungi themselves produce stage-specific ncRNAs to modulate virulence genes involved in protease activity, nutrient acquisition, and hyphal growth. We highlight the dual role of ncRNAs in both promoting host defense and facilitating fungal invasion, linking fungal pathogenicity to immune modulation. Finally, we explore the translational potential of targeting ncRNA networks, outlining a roadmap for novel mechanism-based diagnostics and antifungal therapies against this widespread, recurrent infection.

## Introduction

Fungal infections pose a substantial threat to public health worldwide due to the ubiquitous presence of fungi in ecosystems, including soil, organic matter, and living organisms such as mammals and insects. Among fungal pathogens, dermatophytes – including *Trichophyton rubrum* (*T. rubrum*), *Trichophyton mentagrophytes* (*T. mentagrophytes*), and *Microsporum canis* (*M. canis*) – constitute the most prevalent etiological agents of keratinophilic infections [1]. These filamentous fungi selectively colonize keratin-rich tissues (skin, hair, and nails), causing dermatophytosis – a condition affecting 20–25% of immunocompetent individuals globally and ranking as the fourth most common cutaneous infection [2]. The pervasive nature of dermatophytosis imposes substantial strain on healthcare infrastructures worldwide.

The initial step in dermatophyte infection involves breaching the skin physical, chemical, and microbial barriers through enzymatic and mechanical disruption. Subsequent pathogenesis progresses through three sequential phases: (1) Adhesion: Surface adhesins mediate binding to epidermal keratinocytes (KCs); (2) Hyphal proliferation: Metabolic reactivation under permissive conditions drives mycelial expansion; (3) Keratinolysis: Secreted keratinases (e.g. subtilisins and metalloproteases) degrade keratin into oligopeptides and amino acids, which are assimilated for fungal growth [3]. Native keratin’s structural resilience arises from intra- and interchain disulfide crosslinks forming a three-dimensional matrix [4]. Dermatophytes employ sulfitolysis to cleave these disulfide bonds, followed by proteolytic hydrolysis to complete keratin digestion [2,5]. Host defense mechanisms engage both innate and adaptive immune components. KCs, Langerhans cells, dendritic cells (DCs), and neutrophils recognize fungal pathogen-associated molecular patterns (PAMPs) via pattern recognition receptors (PRRs), initiating antifungal responses such as phagolysosomal degradation, reactive oxygen species (ROS) production, and cytokine-mediated inflammation [2].

NcRNAs, constituting more than 90% of the human transcriptome, orchestrate cellular processes through epigenetic, transcriptional, and post-transcriptional regulation [6]. Emerging evidence highlight roles for ncRNAs, such as miRNAs, lncRNAs, circRNAs, transfer RNA (tRNA), and tRFs, in modulating fungal virulence and host-pathogen crosstalk. Elucidating ncRNA-mediated regulatory networks offers transformative potential for antifungal therapy. Targeting these molecules could enable precision interventions to disrupt fungal pathogenicity while enhancing host defense fidelity. This review synthesizes current advances in dermatophyte biology, cutaneous immunity, and ncRNA functionality, proposing novel therapeutic strategies to accelerate the development of cost-effective, mechanism-driven antifungals.

## Pathogenic mechanisms of Dermatophytosis

Following epidermal adherence, dermatophytes undergo hyphal extension and conidial germination to anchor within the stratum corneum. These fungi secrete an array of virulence-associated proteins that serve dual roles: (1) degrading structural components of host tissue, and (2) modulating cutaneous immune responses through PRR activation. Key pathogenic effectors include keratinolytic enzymes (e.g. subtilisin-like proteases, metalloproteases) facilitating tissue invasion, and immunogenic cell wall components (e.g. β-glucans, mannoproteins) recognized by C-type lectin receptors on antigen-presenting cells (APCs). Our review focuses on three predominant dermatophyte species: *T. rubrum*, *T. mentagrophytes*, and *M. canis*, elucidating their distinct yet overlapping pathogenic strategies.

As we examine each species in turn (Pathogenic mechanisms of *T.* *rubrum*-2.3), three overarching themes emerge, which will be revisited in the critical appraisals: (1) the hierarchy of evidence – most mechanistic insights derive from *in vitro* models, with limited *in vivo* validation; (2) species-specific adaptations – the chronic course of *T. rubrum*, the acute inflammation of *T. mentagrophytes*, and the zoonotic transmission of *M. canis* reflect distinct virulence strategies; and (3) knowledge gaps – the relative contribution of individual virulence factors to clinical outcomes remains poorly defined for all three species.

### Pathogenic mechanisms of *T.* *rubrum*

The *T. rubrum* species complex represents the predominant etiological agent of human dermatophytosis, accounting for approximately 80–90% of chronic dermatophytosis cases globally [7,8]. This anthropophilic complex comprises three phylogenetically distinct taxa – *T. rubrum*, *T. violaceum*, and *T. soudanense* – which are distinguishable by multi-gene analyses [9,10]. Morphologically, they share hyphae, microconidia, arthroconidia, macroconidia, chlamydospores, and zygospores; pycnidia have been identified as key virulence-associated propagules [11]. Notably, *T. violaceum* lacks microconidia and forms cream colonies, whereas *T. soudanense* and *T. rubrum* produce abundant microconidia with yellow-orange and brown pigmentation, respectively [12].

Clinically, *T. rubrum* demonstrates broad tissue tropism, capable of colonizing glabrous skin (manifesting as tinea corporis, tinea pedis, tinea cruris, and tinea manuum), pilose follicles (tinea capitis), and nail units (onychomycosis) even in immunocompetent hosts [13]. Onychomycosis caused by *T. rubrum* exhibits characteristic dermoscopic features including spikes and ruin pattern, with white discoloration showing 100% specificity for *T. rubrum* infection [13]. Although primarily superficial, this species can invade deeper tissues and disseminate systemically. The clinical course is typically subacute, chronically progressive, and prone to recurrence, posing therapeutic challenges. In contrast, *T. violaceum* and *T. soudanense* predominantly cause tinea capitis in endemic regions, while *T. rubrum* favors cutaneous and ungual sites – a tropism linked to differential keratinase expression across anatomical niches [14]. Moreover, the emergence of antifungal-resistant *T. rubrum* strains, particularly terbinafine-resistant isolates harboring *SQLE* gene mutations, poses an emerging challenge for clinical management [15].

Elucidating the molecular mechanisms underlying this pathogenic versatility requires detailed understanding of the infection process, which encompasses three critical phases: tissue adhesion, keratinolytic colonization, and enzymatic penetration [16].

#### Adhesion and invasion mechanisms

**Figure 1.**
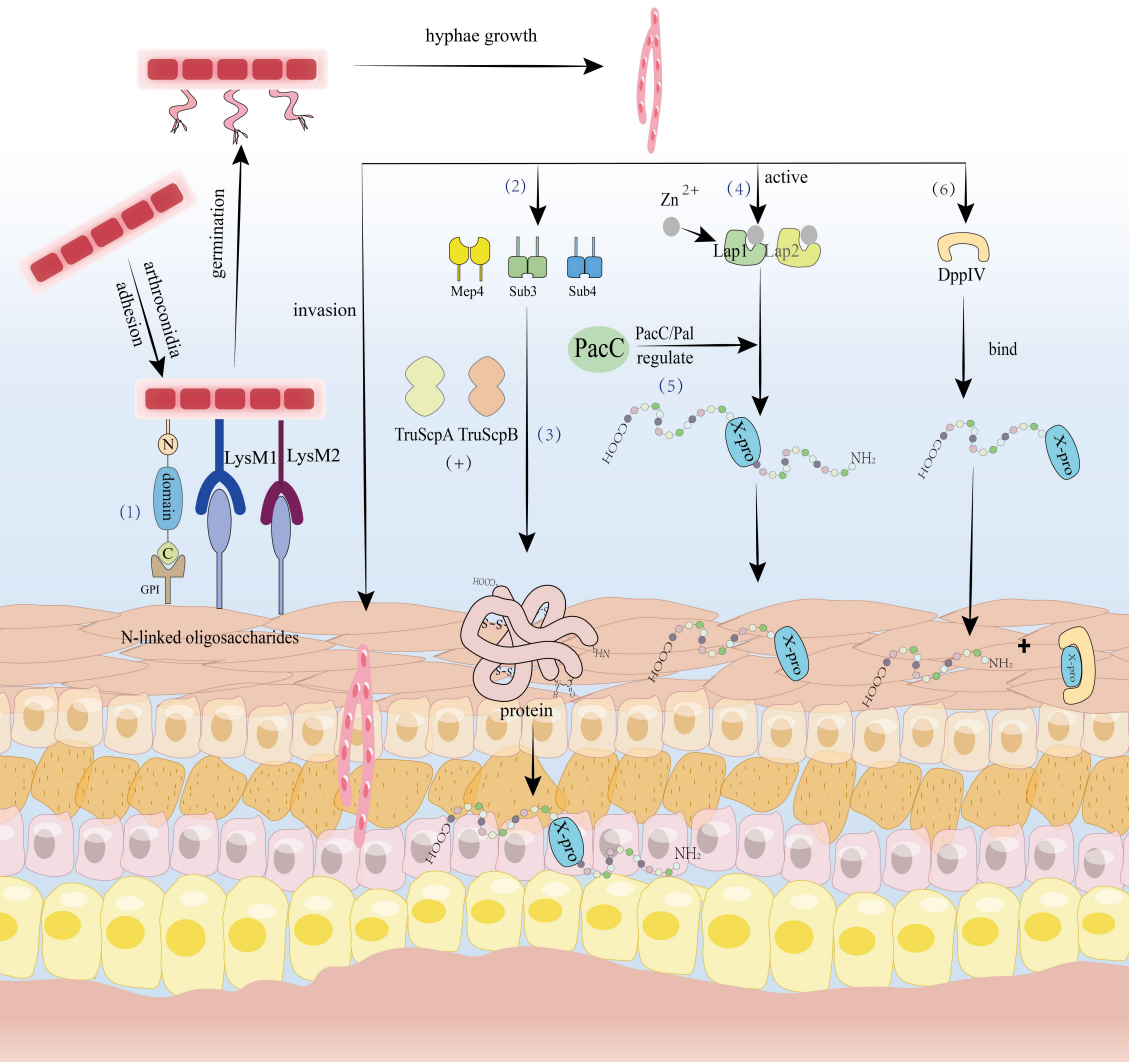
Pathogenic mechanisms of *T. rubrum*.

Host adhesion is mediated through surface-expressed carbohydrate-binding adhesins localized on conidial protrusions and interconidial protofibrils as demonstrated in *in vitro* binding assays using human keratinocyte cell lines [17,18]. These modular adhesins feature: (1) an N-terminal ligand-binding domain specific for host glycoconjugates, (2) a central Ser/Thr-rich tandem repeat region conferring structural flexibility, and (3) a C-terminal Glycosylphosphatidylinositol (GPI)‑anchor domain enabling covalent integration into the fungal cell wall based on bioinformatic predictions [19] (Figure 1). Complementing this, *T. rubrum* secretes LysM-type effector proteins (LysM1/2) that bind to N-linked oligosaccharides on host skin glycoproteins, thereby enhancing fungal adherence to epidermal matrices and promoting adhesion to human skin, as shown by in vitro binding assays using human skin lysate [20] (Figure 1). Recent functional studies identify TrCla4, a p21-activated kinase (PAK), as a critical regulator of hyphal tip actin polymerization that drives hyphal growth and tissue invasion, with *in vitro* knockout in *T. rubrum* confirming its essential role in spore germination and hyphal elongation [21].

(1) Conidial surface adhesins are GPI-anchor for cell wall attachment, facilitating spore adhesion to skin. LysM1 and LysM2 further enhance attachment by binding to N-linked oligosaccharides on host glycoproteins. (2) *T. rubrum* secretes endoproteases Sub3, Sub4, and Mep4, which hydrolyze proteins into X-Pro dipeptide-containing oligopeptides. (3) TruScpA and TruScpB are GPI-anchored serine carboxypeptidases that bind to keratinocyte membranes and synergize with endoproteases, enabling targeted proteolysis of host structural proteins and enhancing pathogenicity. (4) Lap1/Lap2 are Zn^2+^ -dependent aminopeptidases that mediate progressive N-terminal degradation until encountering an X-Pro motif, where steric hindrance halts proteolysis. (5) The PacC/Pal signaling pathway enables rapid adaptation to pH fluctuations, optimizing Lap1/Lap2 activity. (6) Secreted DppIV cleaves the X-Pro motif, re-exposing the N-terminus for continued proteolytic processing.

#### The proteolytic cascade

Following successful adhesion, quiescent arthrospores germinate into invasive hyphae that deploy a hierarchical protease system. This system operates through a bipartite enzymatic cascade, initiated by subtilisin-like endoproteases (Sub3, Sub4, Mep4) that cleave conserved structural motifs in keratin, yielding 15–30 kDa oligopeptides bearing terminal X-Pro dipeptide sequences, as demonstrated by *in vitro* biochemical assays with purified enzymes and synthetic keratin substrates [22,23] (Figure 1). Subsequent processing involves coordinated action of M28-family metallopeptidases (leucine aminopeptidases Lap1/Lap2) and S9B-subfamily serine proteases (dipeptidyl peptidases DppIV/DppV). Lap1/Lap2 mediate progressive Zn^2+^ -dependent N-terminal degradation until steric hindrance from proline’s pyrrolidine ring in X-Pro sequences halts proteolysis, whereupon DppIV/DppV excise these restrictive motifs via catalytic triad (Ser-Asp-His)-mediated β-proteolysis, thereby regenerating accessible N-termini for continued substrate catabolism based on *in vitro* enzymatic assays [22,23] (Figure 1).

In addition to secreting various proteases, *T. rubrum* produces a metallocarboxypeptidase (TruMcpA) and two membrane-anchored serine carboxypeptidases (TruScpA and TruScpB) when cultured in protein-based nitrogen and carbon media [24]. These two GPI-anchored enzymes bind to membranes and synergize with endoproteases and aminopeptidases during infection, enhancing virulence by breaking down keratinized tissues into absorbable amino acids and peptides [25] (Figure 1). Beyond proteases, *T. rubrum* expresses a repertoire of additional virulence factors including lipases, phospholipases, hemolysins, and elastases. *In vitro* phenotypic characterization of clinical isolates revealed lipase expression levels reaching high to very high levels [26].

#### pH-dependent regulation

The efficiency of this proteolytic machinery is further modulated by environmental pH, to which *T. rubrum* exhibits remarkable adaptability. Enzymatic function is pH-dependent; for instance, Lap1 and Lap2 remain active over a broad range (pH 6.5–10.5), with maximal activity between pH 7.0 and 9.0 [23]. Additionally, *T. rubrum* can rapidly adapt to pH fluctuations by regulating gene expression to optimize enzyme functionality [27]. This pH-responsive mechanism, which contributes to fungal virulence, relies on the PacC/Pal signaling pathway. In this pathway, PacC – a transcriptional regulator – modulates pH-dependent gene expression [27,28] and is activated through proteolytic cleavage triggered by a signaling cascade involving six Pal proteins (palA, palB, palC, palF, palH, palI) that sense alkaline conditions [29–31] (Figure 1). Notably, treatment with hsp90 inhibitors reduces pacC transcript levels in keratin cultures, suggesting a functional relationship between hsp90 and PacC that may influence fungal virulence [32].

#### Critical appraisal of *T.* *rubrum* studies

While the enzymatic components of the keratinolytic machinery have been extensively cataloged, several limitations warrant consideration. First, most studies are descriptive (identifying which proteases are expressed) rather than functional (demonstrating necessity through genetic knockout or inhibition). Second, the majority of findings derive from *in vitro* cultures; the relevance of specific proteases to human infection has not been systematically validated. Third, redundancy in the protease arsenal may compensate for loss of any single enzyme, complicating functional interpretation. Fourth, while the PacC/Pal pathway is elegantly characterized biochemically, its *in vivo* importance during infection has not been tested in animal models.

### Pathogenic mechanisms of T. mentagrophytes

The *T. mentagrophytes* complex is the second most common cause of dermatophytosis worldwide (15–25% of cases), following *T. rubrum* [33]. It comprises five species—*T. mentagrophytes*, *T. interdigitale*, *T. erinacei*, *T. quinckeanum*, and *T. benhamiae*—alongside nine genotypic variants and hybrids of *T. mentagrophytes*/*T. interdigitale* [34,35]. Of particular concern is the emergence of terbinafine-resistant *T. indotineae* (formerly genotype VIII), which now shows transcontinental spread [36,37]. This multidrug-resistant pathogen exhibits high clinical persistence in tinea corporis, cruris, and faciei cases, with terbinafine resistance mediated by both *in vitro* genetic mutations and *in vivo* therapeutic failure [38]. In regions such as Iran, *T. indotineae* has become the dominant dermatophyte, accounting for up to 76% of all dermatophytosis cases, with *SQLE* mutations (primarily F397L and L393S) detected in 45% of isolates [39].

*T. mentagrophytes* is predominantly zoophilic (75.9–77.5% prevalence), with primary reservoirs including companion animals (guinea pigs, rabbits) and fur-bearing wildlife (foxes, minks). Human infections arise through direct/indirect animal contact, manifesting as tinea corporis, tinea cruris, tinea barbae, and tinea faciei based on recent surveillance data [40].

#### Adhesion and invasion mechanisms

**Figure 2.**
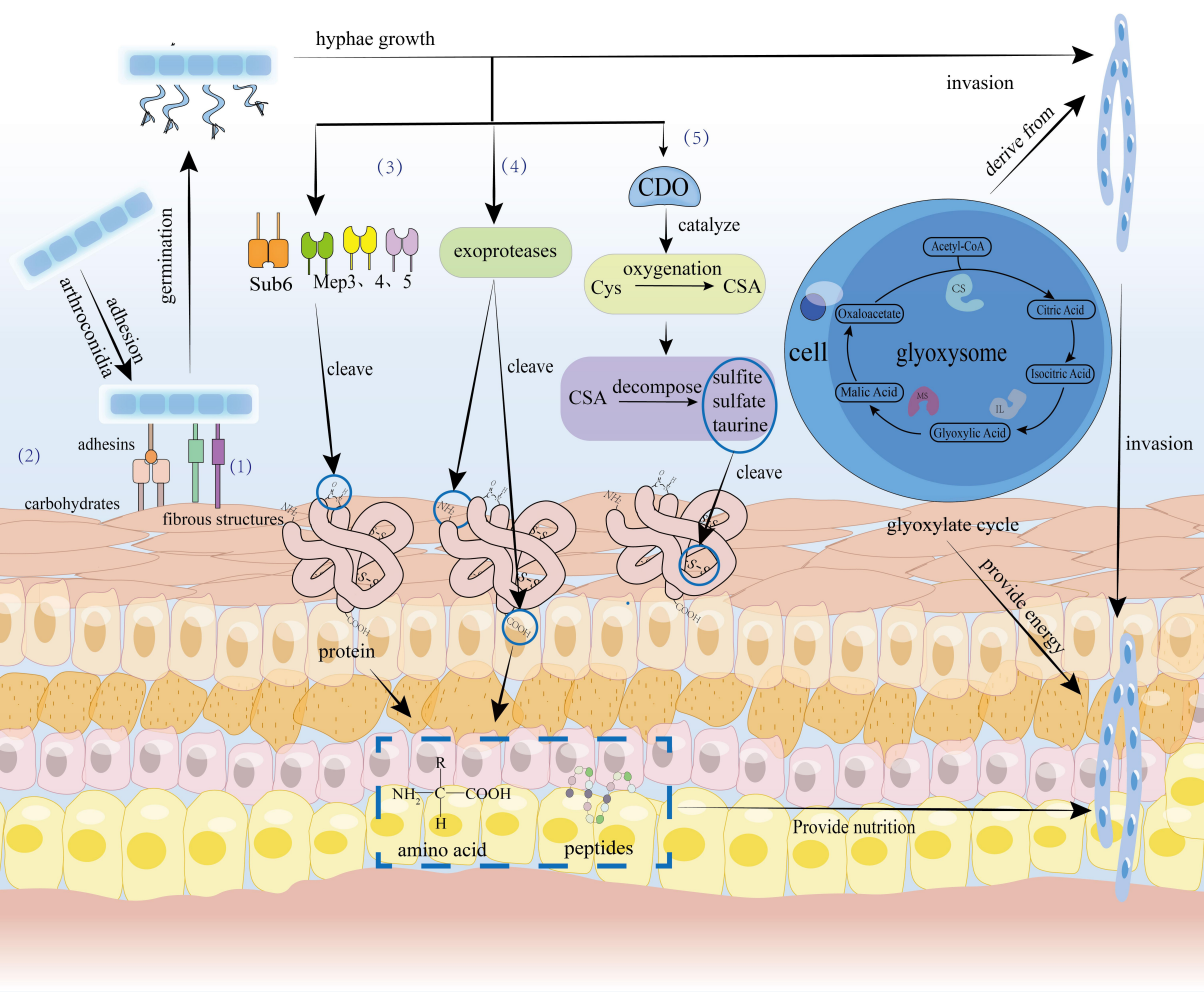
Pathogenic mechanisms of *T. mentagrophytes*.

The pathogenesis of *T. mentagrophytes* involves four sequential stages: adhesion, budding, penetration, and tissue invasion. *In vitro* assays with skin explants showed that arthrospores adhere to the stratum corneum within 6 h of contact and anchor to keratinized tissues via a dual system of elongated and truncated fibers, without disrupting keratinocyte membranes, as revealed by ultrastructural analysis [41] (Figure 2). More recent studies using reconstructed human epidermis models have demonstrated that adhesion can occur as early as 30 minutes post-contact, with the subtilisin protease Sub6 playing a key role during this initial adhesion phase [42,43].

(1) Arthrospores adhere to keratinized tissues via elongated and truncated fibrous structures. (2) Arthroconidia express surface adhesins for specific carbohydrate recognition. (3) Major secreted endopeptidases (MEP3/4/5 and Sub6) cleave internal peptide bonds. (4) Exopeptidases hydrolyze peptide bonds at N- or C-termini. (5) CDO catalyzes cysteine oxygenation, generating sulfite, sulfate, and taurine, reducing disulfide bonds (–S–S–) in keratin to facilitate fungal invasion. (6) Glyoxylate cycle enzymes are implicated in pathogenesis, though their precise roles require further elucidation.

Notably, adhesion mechanisms exhibit interspecies variation. In *T. interdigitale* (a species within the *T. mentagrophytes* complex), germinating conidia utilize protofibrillar extensions from their outer cell walls to establish primary contact with palmar KCs. Subsequent hyphal branching and secondary germ tube formation further reinforce attachment to adjacent KCs [18], and *ex vivo* adhesion assays reveal a time-dependent increase in spore adhesion density with prolonged contact [44]. *In vitro* binding assays show that *T. mentagrophytes* arthroconidia express surface adhesins that bind mannose and galactose, implicating these lectin-like proteins in initial host-pathogen adhesion [45] (Figure 2).

#### Hyphal invasion and proteolytic arsenal

After germination, *T. mentagrophytes* germ tubes elongate and penetrate the stratum corneum using physical force and secreted hydrolases, establishing intradermal infection foci in vitro skin explant models [46] (Figure 2). Although hyphal branches adhere superficially to keratinized layers, they exhibit lower adhesive capacity than spores [46].

Functionally, invasive hyphae deploy a sophisticated enzymatic arsenal, particularly keratinolytic proteases, to achieve coordinated enzymatic degradation of structural keratin. This proteolytic processing yields low-molecular-weight peptides and free amino acids for fungal uptake. Importantly, these nutritionally assimilated keratin derivatives serve both as ATP-generating substrates and as biosynthetic precursors, thus sustaining the pathophysiological progression of dermatophytosis [47]. *In vitro* quantitative real-time PCR (qRT-PCR) analysis revealed that *T. mentagrophytes* predominantly transcribes the genes encoding the endoproteases MEP3, MEP4, MEP5 (metalloproteases), and Sub6, which cleave peptide bonds within polypeptides [48] (Figure 2). Agrobacterium tumefaciens-mediated transformation (ATMT)-based functional characterization shows that MEP3 possesses the strongest *in vitro* proteolytic activity and *ex vivo* hair degradation, whereas MEP4 and MEP5 are least pathogenic *in vivo*, highlighting their key role in *T. mentagrophytes* virulence [49]. Sub6, a major allergen, elicits both immediate and delayed DTH responses, and Sub6 disruption *in vivo* attenuated clinical symptoms while upregulating MEP4 and Sub3, revealing regulatory interplay among Sub6, MEP4, and Sub3 [50]. Recent genomic analysis of clinical *T. mentagrophytes* isolates revealed multiple virulence factors (MEP-1 to MEP-5) and clonal relatedness between Italian and Chinese strains [33]. Following endoprotease cleavage, exoproteases further degrade polypeptides by cleaving peptide bonds from either the N-terminus or the C-terminus (Figure 2). In addition to these proteases, *T. mentagrophytes* also secretes cysteine dioxygenase (CDO, EC 1.13.11.20), which catalyzes the conversion of cysteine to cysteine sulfinic acid, yielding sulfite, sulfate, and taurine that reduce disulfide bonds in host keratins [51] (Figure 2).

Furthermore, studies have shown that non-proteolytic enzymes of the glyoxylate cycle, such as citrate synthase (CS), isocitrate lyase (IL), and malate synthase (MS), play a role in *T. mentagrophytes* infection, as demonstrated by qRT-PCR analysis of *in vitro* cultured fungi (Figure 2). The specific mechanisms through which these enzymes function require further exploration [48]. Zinc is critical for *T. mentagrophytes* growth, as *in vitro* zinc limitation markedly suppressed proliferation [52]. Transcriptome sequencing identified 10,751 unigenes, including the zinc-responsive transcriptional activator ZafA and four candidate zinc transporters [52]. Targeted disruption of ZafA (ZafA-hph) reduced fungal growth, reproduction, zinc uptake, *ex vivo* hair degradation, and *in vivo* pathogenicity relative to the wild-type and complemented strain [53]. Collectively, these data demonstrate that ZafA functions as a positive regulator of zinc homeostasis and is indispensable for the pathogen’s full virulence [52,53].

#### Critical appraisal of *T.* *mentagrophytes* studies

Several caveats warrant consideration. First, the reported 30-minute adhesion kinetics derive from reconstructed human epidermis models; whether this rapid adhesion occurs in *in vivo* human infection remains to be confirmed. Second, conflicting results exist regarding the precise timing of adhesion across different experimental systems (traditional vs reconstructed epidermis models), possibly due to differences in fungal strain preparations or detection sensitivities. Third, while the enzymatic arsenal of *T. mentagrophytes* has been well cataloged through *in vitro* studies, functional validation through genetic eknockout (e.g. Sub6, MEP4/5) in animal models is limited. Fourth, the emergence of terbinafine-resistant *T. indotineae* is well-documented epidemiologically, but the fitness costs associated with *SQLE* mutations and the clinical implications for treatment outcomes require further investigation.

### Pathogenic mechanisms of *M.* *canis*

The genus *Microsporum* encompasses three phylogenetically distinct dermatophytes—*M. canis*, *M. ferrugineum*, and *M. audouinii*—which exhibit differential pathogenic profiles across humans and animals [54–56]. Comparative genomic analysis has confirmed high conservation of nuclear and mitochondrial genomes among these three species, despite their distinct host preferences [57]. Phylogenetic analyses place *M. canis* as the earliest-diverging dermatophyte lineage, a geophilic-to-zoophilic transitional species that evolved from soil-associated ancestors to feline hosts [58,59].

*M. canis* is a major pathogenic dermatophyte in domestic animals with zoonotic potential [60,61]. Transmission occurs via anthropozoonotic, reverse zoonotic, and environmental routes. It causes dermatophytosis in felids and canids. In immunocompromised humans, keratinolytic activity facilitates invasion, leading to tinea capitis, onychomycosis, and extensive tinea corporis [62–64].

Whole-genome sequencing of canine/feline *M. canis* reveals a ~ 22.8-Mb genome (47.4% GC), 158 predicted carbohydrate-active enzyme genes linked to virulence, and ANI > 99.9% with human invasive strains, suggesting diverse genotypes with high zoonotic potential [56]. As observed using different *in vitro* and *ex vivo* experimental models, *M. canis* mediates adhesion and invasion through hyphal production. During this process, spore germination generates germ tubes that develop into hyphae, which subsequently produce asexual conidia critical for initiating infection [45,65]. These conidia adhere to keratinized tissues, germinate into hyphae, and form hyphal colonies via tip growth and branching. During infection, invasive filamentous hyphae penetrate skin both longitudinally and vertically, exacerbating tissue damage as demonstrated using *in vitro* hair models [45,66].

#### Adhesins and morphogenesis

**Figure 3.**
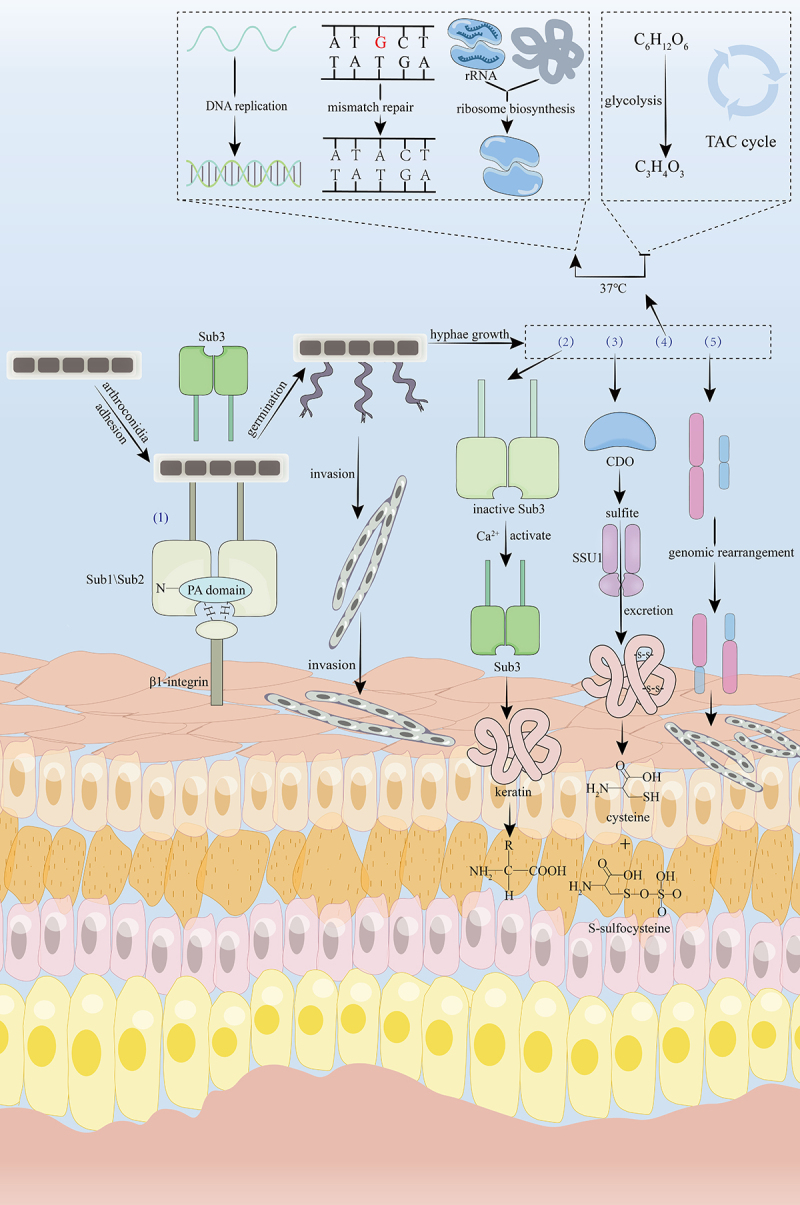
Pathogenic mechanisms of *M. canis.*

The pathogenicity of *M. canis* is mediated through specialized arthroconidial adhesins, particularly Sub3, which is required for adherence as demonstrated by *in vitro* assays and *ex vivo* epidermal models [65,67,68]. *In vitro* inhibition experiments confirmed lectin-mediated binding mechanisms [51], while *in vivo Sub3* gene silencing revealed that Sub3 is required for adherence but not for epidermal invasion [65] (Figure 3). *Ex vivo* adherence assays demonstrated that *Sub3* silencing significantly reduces fungal adherence across species [68]. DPP IV activity is involved in adhesion and early invasion [69], and Sub3 is highly prevalent in clinical isolates [70]. FSH1, a serine esterase, regulates conidial morphogenesis and virulence; its knockout impairs septation and attenuates pathogenicity [71]. Notably, comparative genomics further suggests that expansions of MFS transporters and Zn_2_Cys_6_ transcription factors in anthropophilic species *M. audouinii* and *M. ferrugineum* may facilitate host adaptation relative to zoophilic *M. canis* [57].

(1) Sub3-mediated adherence: Sub3 functions as a surface adhesin via lectin-like recognition and is required for adherence. Sub1/Sub2 keratinase anchoring: N-terminal PA domains target host β1-integrin, inducing conformational fixation and aligning the catalytic triad for keratinolysis. (2) Sub3 keratinase activation: Ca^2+^ binding stabilizes α-helix clusters, promoting proenzyme-to-active transition. Activated Sub3 degrades keratin and supports hyphal growth/invasion. (3) Sulfite-mediated keratin degradation: CDO generates sulfites; SSU1-mediated efflux drives catalytic sulfitolysis of keratin disulfides into cysteine derivatives. This destabilizes β-sheets, denatures keratin, and creates a thiol-rich microenvironment for proteolysis. (4) Thermal adaptation at 37°C: Prioritizes proliferation via upregulation of DNA replication, mismatch repair, and ribosome biosynthesis, while suppressing catabolic pathways (glycolysis, TCA cycle). (5) Genomic rearrangements: Drive chronic infection and deep tissue invasion by selectively upregulating genes for iron acquisition, immune evasion (N-glycan biosynthesis), and proliferation.

#### Proteolytic virulence arsenal

The proteolytic virulence arsenal of *M. canis* encompasses multifunctional elastolytic, collagenolytic, and keratinolytic enzymes capable of processing structural proteins into immunomodulatory oligopeptides, with their tripartite functionality spanning host matrix adhesion, stratum corneum invasion, and immune evasion based on *in vitro* biochemical characterization [54,72]. Mechanistically, Sub1/Sub2 employ their N-terminal PA domains to anchor to the host surface via β1-integrin recognition, while Sub3 undergoes conformational activation through Ca^2+^ -dependent α-helix stabilization [73,74] (Figure 3). Importantly, Sub3 facilitates immune subversion through the degradation of TLR4 ligands, though its mechanisms of latency establishment remain unknown [65,73].

In *Aspergillus* spp., protease gene expression is induced by protein-rich conditions via the nitrogen regulator AreA; similarly, in *M. canis*, AreA inactivation reduces protease expression, proteolytic activity, and keratin-based growth, as confirmed by *in vitro* knockout studies [75]. *M. canis* also secretes CDO, which catalyzes cysteine oxygenation to sulfite (confirmed by knockout [3,4]), and then utilizes SSU1‑mediated sulfite efflux to induce sulfitolysis of cystine disulfides into cysteine and S‑sulfocysteine [3,5,76,77], thereby denaturing keratin via β‑sheet destabilization and creating a thiol‑rich environment for proteolysis [3,22,78] (Figure 3).

#### Invasive strategies

The invasive pathogenicity of *M. canis* involves host resource exploitation, thermal adaptation, and genomic evolution. Transcriptomic analysis of virulent strains (e.g. BMU08102, BMU08359) revealed upregulation of membrane transporters (for sugars, phosphate, and calcium) and iron/heme-acquisition pathways, enhancing nutrient scavenging [79]. This may mimic Candida albicans in extracting heme from host hemoglobin to facilitate dermal and vascular invasion [80]. At host body temperature (37°C), they prioritize proliferation via upregulated DNA replication, mismatch repair, and ribosome biosynthesis pathways while suppressing metabolic activity – adopting a “high-growth, low-metabolism” strategy to circumvent thermal susceptibility (Figure 3). Genomic rearrangements further enhance virulence by selectively upregulating genes involved in iron acquisition, immune evasion (e.g. N-glycan biosynthesis), and proliferation, collectively enabling chronic infection and deep tissue invasion [79] (Figure 3).

#### Critical appraisal of *M.* *canis* studies

While *M. canis* has been less intensively studied than *T. rubrum* or *T. mentagrophytes*, recent genomic and functional studies have substantially advanced our understanding. However, several limitations persist. First, most functional studies (e.g. Sub3 knockout) have been performed in feline epidermis models; validation in human skin models or *in vivo* animal models is limited. Second, the proposed “high-growth, low-metabolism” thermal adaptation strategy is derived from transcriptomic data; direct metabolic flux measurements are lacking. Third, the clinical significance of the diverse genotypes identified by comparative genomics (ANI > 99.9%) remains unclear – whether specific genotypes correlate with increased virulence, transmission efficiency, or treatment outcomes has not been established in clinical studies. Fourth, comparative studies between *M. canis* and other *Microsporum* species (*M. ferrugineum*, *M. audouinii*) are virtually non-existent, leaving open the question of which virulence features are unique to *M. canis* vs conserved across the genus.

Functional assays have demonstrated that anthropophilic *Microsporum* strains exhibit higher tolerance to acidic pH and enhanced keratinase activity in lipid-rich environments compared to zoophilic strains, with *M. ferrugineum* showing the strongest osmotic tolerance [57]. These observations provide phenotypic validation for the genomic differences observed between anthropophilic and zoophilic species.

### Summary comparison across species

The three species exhibit distinct pathogenic profiles across several clinically relevant dimensions. (1) Host range and ecology: *T. rubrum* is strictly anthropophilic; *T. mentagrophytes* is zoophilic with soil saprophytic survival; *M. canis* is zoonotic with tripartite transmission. (2) Clinical course: *T. rubrum* causes chronic, relapsing infections with broad tissue tropism (notably nails); *T. mentagrophytes* typically elicits acute inflammatory responses; *M. canis* frequently causes tinea capitis in children. (3) Protease repertoire and molecular mechanisms: While all three secrete subtilisin-like proteases and metalloproteases, species-specific differences exist (e.g. Sub6 prominence in *T. mentagrophytes*, CDO conservation across species). It remains unclear whether differences in protease profile, cell wall composition, or immunomodulatory capacity underlie their divergent pathogenesis. Notably, the pH-regulatory PacC/Pal pathway, which influences virulence, has been best characterized in *T. rubrum*; whether *T. mentagrophytes* and *M. canis* employ analogous mechanisms is unknown. (4) Antifungal resistance: Terbinafine resistance is emerging in *T. rubrum* (*SQLE* mutations) and *T. indotineae* (a lineage within the *T. mentagrophytes* complex), but has not been reported for *M. canis*. (5) Genomic resources and research bias: *M. canis* genomics has advanced recently with whole-genome sequencing, but functional validation lags behind *T. rubrum*. Whether the observed pathogenic differences reflect true biological distinctions or are artifacts of differential study intensity – given that *T. rubrum* has been far more extensively studied – remains a key open question.

## Skin resistance mechanisms

Dermatophytes exploit host proteins via keratinolytic activity. Although restricted to superficial tissues, these infections elicit innate, humoral, and cellular immune responses through the skin immune system [2]. Having established the virulence arsenals of the three major dermatophyte species, we now examine the host side of the host-pathogen equation. The skin is not a passive target but an active immune organ that deploys multilayered defenses – from antimicrobial peptides to adaptive immunity – against fungal invasion. Understanding these host resistance mechanisms is essential for appreciating how dermatophytes, despite their virulence capabilities, are normally kept in check, and how ncRNAs may modulate the outcome of infection.

### Skin innate immunity and dermatophytosis

The skin and mucous membranes constitute the primary physical barrier of the skin immune system against fungal infections, synthesizing antimicrobial substances through epidermal cells. Upon breaching this barrier, dermatophytes trigger a coordinated response involving effector cells, chemokines, and AMPs to eliminate pathogens and restore skin integrity. This process includes immune recognition of fungi, accelerated regeneration of the epidermis and KCs, and the release of inflammatory factors to clear infection.

#### AMPs and dermatophytosis

AMPs – including defensin and cathelicidin families – serve as key innate immune effectors in skin defense, with diverse types such as human β-defensins (hBD), LL-37 (cathelicidin), S100 proteins, and ribonuclease 7, exhibiting elevated expression in *T. rubrum*-infected epidermis as shown in clinical biopsy samples [81–83]. *In vitro*, skin‑secreted AMPs (e.g. β‑defensin 2, ribonuclease 7, and psoriasin) inhibit the growth of dermatophytes including *M. canis*, *T. rubrum*, and *T. floccosum*, with heightened susceptibility in *T. floccosum*, suggesting that AMPs may specifically counteract fungal invasion [84].

**Figure 4.**
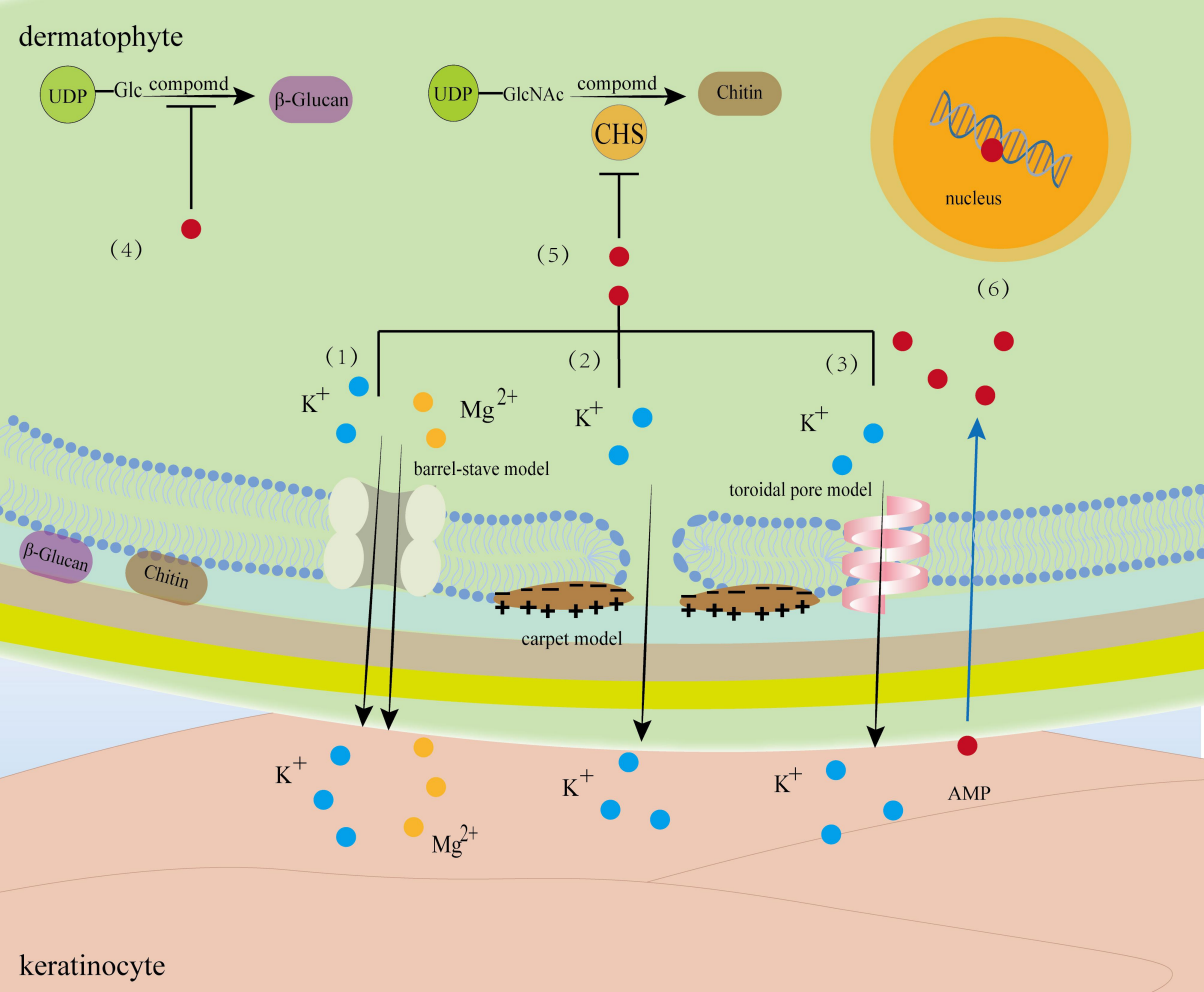
Mechanisms of action of AMPs against dermatophytes.

AMPs against pathogenic fungi can be categorized into two groups based on their distinct mechanisms of action. The first group comprises membrane-targeting AMPs, commonly termed pore-forming peptides, which exhibit broad-spectrum antifungal and antibacterial activity by disrupting microbial membranes through three primary models. In the barrel-stave model, aggregated peptides form barrel-shaped pores that perforate fungal membranes, causing rapid potassium/magnesium efflux and cell death [85] (Figure 4). The carpet model involves electrostatic accumulation of AMPs on membrane surfaces, culminating in micelle formation and structural disintegration at high peptide concentrations [86] (Figure 4). The toroidal pore model features peptide insertion that induces lipid layer tilting and toroidal pore formation (Figure 4), exemplified by human LL-37 (murine CRAMP), as demonstrated using *in vitro* membrane model systems [87]. This peptide interacts with fungal cell wall carbohydrates, disrupts membrane integrity, and triggers ROS accumulation that leads to cell death, as shown in *in vitro* fungal culture assays [88].

AMPs act via two primary mechanisms. Membrane-targeting AMPs disrupt membrane integrity through three models: (1) barrel-stave (ion efflux); (2) carpet (membrane disintegration); (3) toroidal pore (circular pore formation). Cell wall-targeting AMPs specifically binds fungal wall components to: (4) inhibit β-glucan synthesis; (5) inhibit chitin cross-linking and promote its degradation; (6) interact with nucleic acids for antifungal effects.

The second AMP category specifically targets fungal cell walls through three specialized mechanisms: β-glucan synthesis inhibition (weakening structural integrity by thinning nascent cell walls), chitin synthase interference (impairing chitin chain extension/cross-linking while activating chitinase-mediated degradation), and nucleic acid binding (exerting antifungal effects through poorly characterized DNA interactions) as shown by *in vitro* enzyme inhibition and binding assays [89] (Figure 4). While significant progress has been made in characterizing these antifungal AMPs, comprehensive elucidation of their molecular mechanisms and therapeutic potential requires further investigation.

#### PRRs recognize dermatophytes

**Figure 5.**
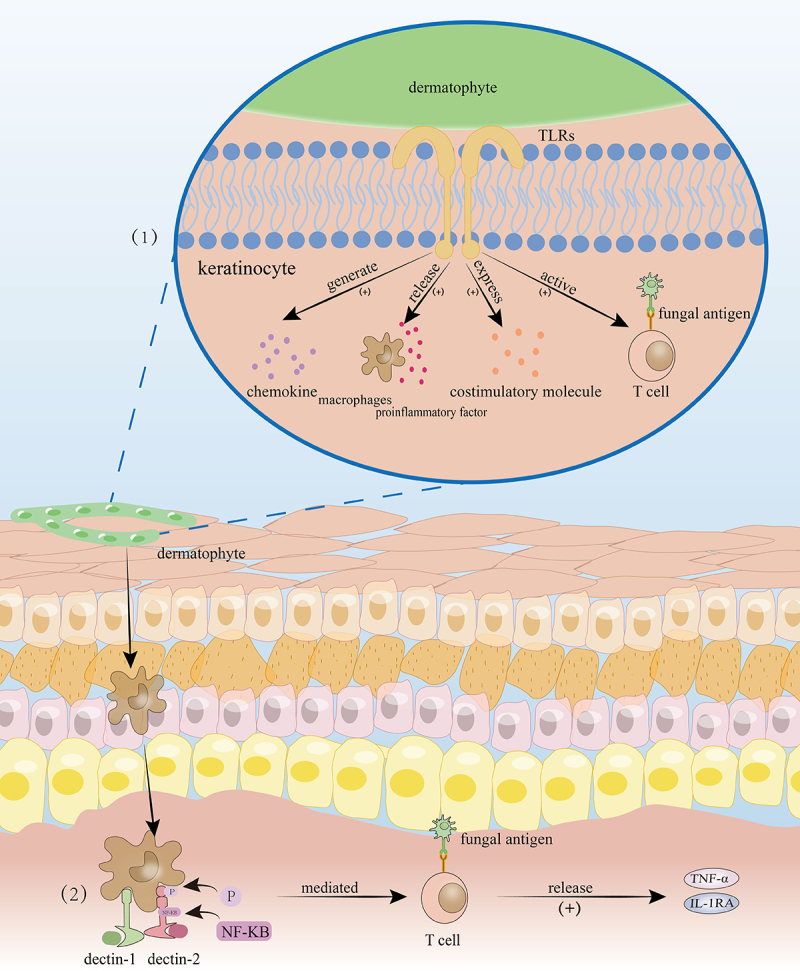
Recognition of dermatophytes by PRRs.

The host immune system has evolved a sophisticated pathogen surveillance network through PRRs that detect conserved microbial structures known as PAMPs. The dermatophyte recognition system involves multiple PRR families including Toll-like receptors (TLRs), nucleotide oligomerization domain-like receptors (NLRs), C-type lectin receptors (CLRs), retinoic acid-inducible gene 1 receptors (RIGs), protease-activated receptors (PARs), and sialic acid-binding immunoglobulin-like lectins (Siglecs) [90]. Among these, TLRs play a pivotal role in fungal recognition and subsequent immune activation, triggering chemokine production, pro-inflammatory factor release, co-stimulatory molecule expression, and adaptive immunity initiation during antigen presentation [91] (Figure 5). Notably, the expression of TLR1 is significantly upregulated post-infection in an *ex vivo* human primary epidermal organoid (hPEO) model, suggesting that it may be involved in orchestrating the cellular immune response against *T. rubrum* infection [92].

(1) TLRs on KCs: Upon fungal recognition, TLRs trigger chemokine production, pro-inflammatory factor release, co-stimulatory molecule expression, and the initiation of acquired immunity during antigen presentation. (2) CLRs (dectin-1/dectin-2): Dectin-1 specifically recognizes β-1,3/β-1,6-glucan to induce TNF-α secretion. Dectin-2 mediates T-cell immune activation via tyrosine phosphorylation and NF-κB activation, inducing TNF-α and IL-1RA secretion (P, phosphorylation).

The CLR family member Dectin-2 mediates immune activation through tyrosine phosphorylation and NF-κB activation upon *T. rubrum* recognition, leading to TNF-α and IL-1RA production as shown in *in vitro* macrophage cultures [93] (Figure 5). *T. rubrum* induces host release of IL-1RN (interleukin-1 receptor antagonist), which impairs pro-inflammatory IL-1 signaling and suppresses skin inflammation, thereby facilitating immune evasion and resistance to host elimination [92]. Notably, coordinated PRR activation during dermatophyte infection triggers robust chemokine production, enabling a highly specific and effective host defense [94].

#### Cellular components, chemokines and dermatophytosis

The skin deploys a multifaceted cellular defense system – comprising NK cells, neutrophils, monocytes, macrophages, DCs, γδ T cells, and KCs – to combat fungal pathogens, with dermatophyte-specific immune dynamics demonstrating unique cellular activation patterns. Although NK cells activated by *Coccidioides paracoccidioides brasiliensis* secrete immunomodulatory cytokines such as IFN-γ and TNF-α to coordinate antifungal responses, clinical studies reveal a significant increase in NK cells and CD14^+^ monocytes in patients with dermatophyte infections, highlighting their crucial role in defending against cutaneous fungal invasion [95]. This dual evidence highlights the adaptability of skin immunity across fungal pathogens, with dermatophyte-driven cellular proliferation offering critical insights into host-pathogen interactions in superficial fungal diseases.

**Figure 6.**
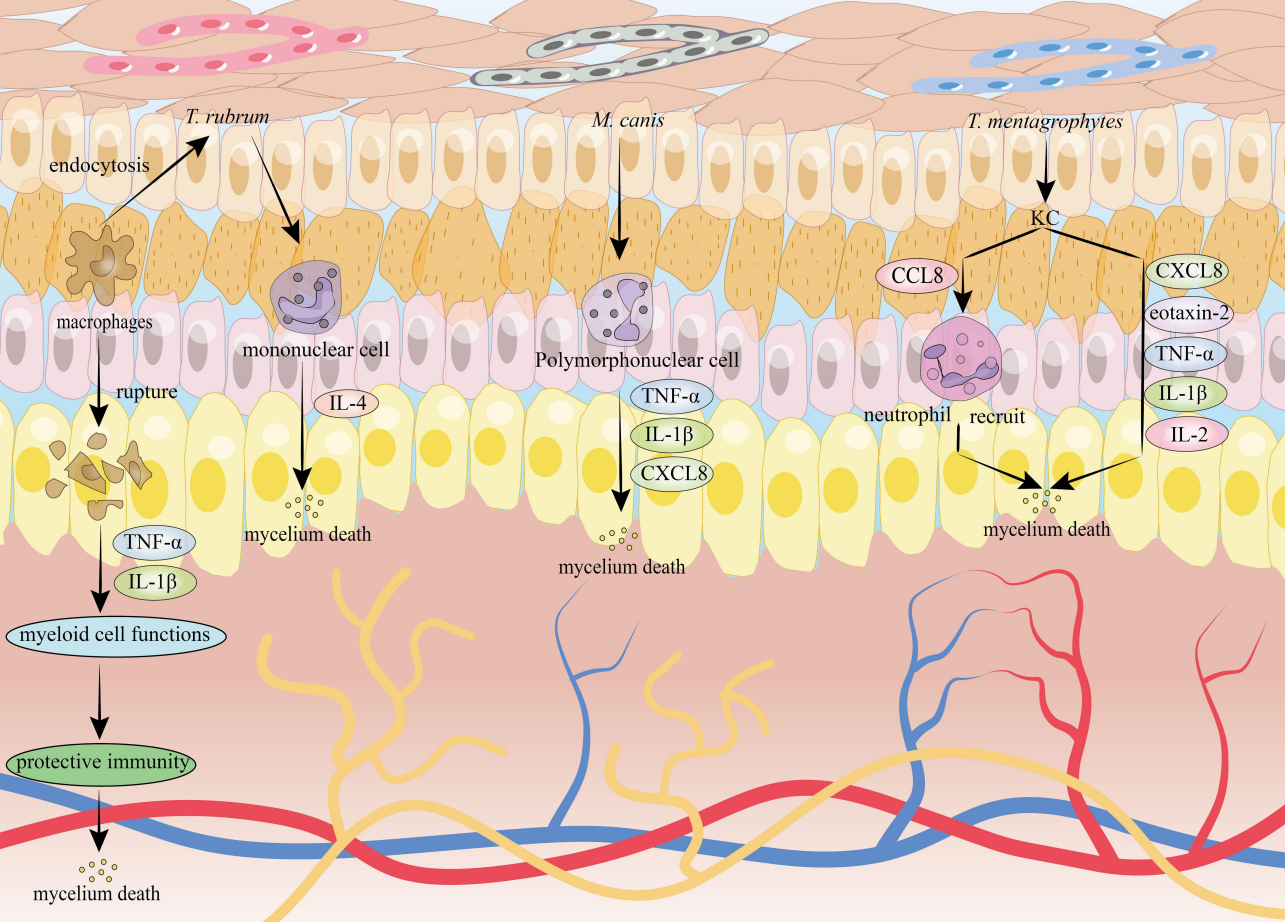
Cellular components, chemokines, and dermatophytosis.

Phagocytes – comprising large phagocytes (monocytes/macrophages) and small phagocytes (neutrophils) – mediate fungal clearance via engulfment, antigen presentation, and cytokine secretion. In *in vitro* co-culture models, peripheral neutrophils exhibit potent cytotoxicity against *T. rubrum* even at an effector: target ratio of 1:1, with maximal fungicidal activity between 2–24 h, declining thereafter; although neutrophils remain viable, they fail to suppress fungal replication beyond 24 h. Furthermore, neutrophil phagocytosis of *T. rubrum* was confirmed by electron microscopy, with disrupted fungal germlings identified within the cytoplasm of phagocytic cells [96]. Macrophages phagocytose *T. rubrum* conidia *in in vitro* phagocytosis assays, yet internalized spores transform into hyphae that rupture phagocytes, a process accompanied by TNF-α and IL-1β production [97]. IL-1β, signaling via IL-1 R, enhances myeloid cell functions (e.g. macrophage phagocytosis, neutrophil recruitment, oxidative burst), suggesting macrophage-mediated phagocytosis triggers protective immunity despite fungal evasion tactics [97,98] (Figure 6). However, *T. rubrum* cell wall mannan inhibits macrophage uptake [97]. Immune dysregulation in atopic dermatitis patients is further evidenced by the release of IL-4 from peripheral blood mononuclear cells upon *ex vivo* stimulation with *T. rubrum* [99]. Polymorphonuclear neutrophils respond to *M. canis* arthrospores by secreting TNF-α, IL-1β, and CXCL8 as shown in *in vitro* neutrophil activation assays [100], underscoring the dual role of these phagocytes in both antifungal defense and inflammation modulation (Figure 6).

Macrophages: Phagocytose *T. rubrum* spores, but hyphal transformation ruptures phagocytes and triggers TNF-α/IL-1β release. IL-1β enhances myeloid cell function to promote antifungal immunity. PBMC: release IL-4 to participate in the immune response and combat *T. rubrum*. PMNs: Combat *M. canis* by releasing TNF-α, IL-1β, and CXCL8, highlighting dual antifungal and pro-inflammatory roles. KCs: Act as physical barriers and immune sentinels, producing cytokines (CXCL8, TNF-α, IL-1β) and chemokines (CCL8) in response to dermatophytes (e.g. *T. mentagrophytes*), recruiting neutrophils and initiating early inflammation – underscoring their pivotal role in host defense.

KCs, the predominant epidermal cells, serve a dual role in dermatophyte infections by constituting a physical barrier and actively participating in immune responses. When infected by dermatophytes, KCs secrete substantial amounts of cytokines and chemokines, including pro-inflammatory mediators. However, the secretory profiles differ markedly between species. *A. benhamiae*-infected KCs exhibit a broad response, secreting multiple proinflammatory, chemotactic, and immunomodulatory factors, whereas *T. tonsurans* triggers only eotaxin-2, IL-8, and IL-16. Microarray analysis further showed that *A. benhamiae* upregulated genes encoding IL-1β, IL-2, IL-4, IL-6, IL-10, IL-13, IL-15, IL-16, IL-17, and IFN-γ, while *T. tonsurans* upregulated only IL-1β and IL-16. Both species enhanced *IL-8* mRNA expression as confirmed by RT-PCR [101]. Studies have demonstrated that *T. mentagrophytes* induces IL-8 and TNF-α production in human keratinocytes [102], with animal-derived isolates eliciting significantly higher IL-8 levels than other dermatophytes [103]. *In vivo* mouse models further confirm increased expression of IL-17A, IFN-γ, and TNF-α following fungal challenge [104], collectively supporting the role of keratinocytes as key responders in the host defense against *T. mentagrophytes* infection. Similarly, fungal components like β-D-glucan from *T. rubrum* and trichophytin from *T. mentagrophytes* induce keratinocytes to secrete CXCL8 and IL-1α in *in vitro* stimulation assays [105]. Additionally, KC-derived CXCL8 facilitates neutrophil recruitment to the stratum corneum [2], demonstrating their crucial role in orchestrating early inflammatory responses against dermatophyte infections (Figure 6).

### Skin adaptive immunity and dermatophytosis

The adaptive immune response to fungal infections involves two primary components: cell-mediated immunity (CMI), which demonstrates antifungal efficacy, and humoral immunity (HI), whose protective role remains debated. While some studies propose antifungal activity in antibodies, inconsistent evidence leaves HI’s functional significance unclear.

#### CMI and dermatophytosis

**Figure 7.**
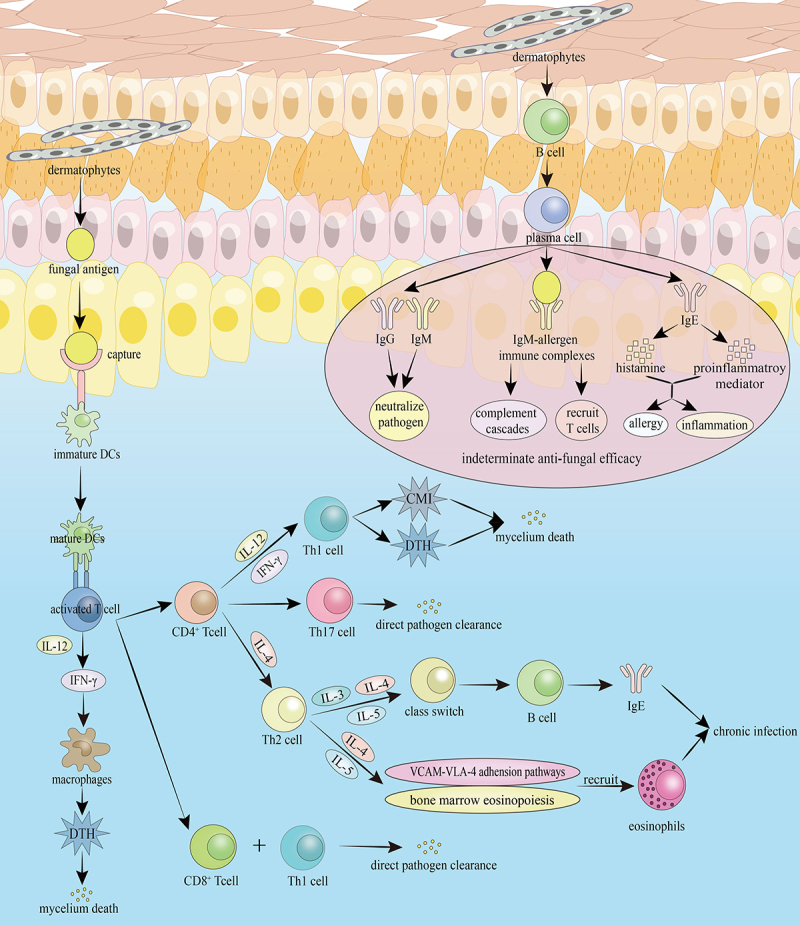
Host CMI and HI responses to fungal antigens.

DCs serve as pivotal initiators and modulators of immune responses, with immature DCs residing in peripheral tissues to capture and present antigens. Upon fungal antigen or cytokine stimulation (e.g. IL-1β, TNF-α), these cells migrate to lymph nodes and spleen, where they activate antigen-specific T cells. During migration, DCs undergo differentiation and maturation, marked by diminished antigen-capturing capacity alongside upregulated expression of MHC II, co-stimulatory molecules (e.g. CD80/86), and adhesion molecules, a process amplified by stimulatory signals that enhance T cell priming efficacy [106] (Figure 7).

Fungal infection triggers DCs to capture antigens, mature, and activate T cells. IL-12 drives Th1 cell development via IFN-γ production, coordinating macrophage-mediated fungal clearance, while Th17 cells directly attack the pathogen. In contrast, IL-4-induced Th2 responses promote susceptibility by stimulating IgE production and eosinophil recruitment, leading to allergic inflammation. CD8^+^ T cells exert direct cytotoxic activity, and B cells produce protective IgG/IgM antibodies, whereas IgE triggers harmful allergic reactions through histamine release. However, the specific antifungal efficacy of HI remains undetermined.

IL-12 acts as a critical inducer of CMI by stimulating IFN-γ secretion from NK and T cells. DTH, a hallmark of CMI against dermatophytes, relies on activated macrophages as terminal effector cells. In DTH, CD4^+^ T cells recognize fungal antigen-MHC II complexes on APCs, triggering pro-inflammatory cytokine release, macrophage recruitment, and fungal antigen-targeted cytotoxicity at infection sites. Recovery from *T. mentagrophytes* infections is regulated by DTH. *In vivo*, Grappel and Blank demonstrated this using a guinea pig cutaneous infection model, in which DTH-positive animals resolved infections and exhibited intense dermal reactivity to keratinase in the absence of detectable circulating antibodies [107]. This DTH response was further confirmed *in vitro* by the inhibition of peritoneal exudate cell migration in response to keratinase [108].

The host immune response to dermatophytes involves dynamic interplay between Th1, Th2, Th17, and CD8^+^ T cell subsets, with Th1/Th17 dominance driving fungal clearance while Th2 polarization correlates with allergic susceptibility. IL-12 and IFN-γ drive CD4^+^ T cell differentiation into Th1 cells, which orchestrate CMI and DTH critical for dermatophyte elimination, while Th17 cells exhibit superior antifungal efficacy in direct pathogen clearance as shown in *in vitro* and *in vivo* studies [109,110]. Th1 cells additionally modulate inflammation resolution by tempering Th17-mediated responses [110]. Complementing these mechanisms, CD8^+^ T cells demonstrate direct cytotoxic activity against dermatophytes alongside Th1 cells [111], with enhanced Th1 proliferation observed in *T. rubrum* infections [112,113] (Figure 7).

Conversely, IL-4-induced Th2 differentiation promotes host vulnerability to dermatophytes through dual pathological pathways: (1) secretion of IL-3/IL-4/IL-5 drives B cell-mediated IgE overproduction via IL-4-stimulated class switching, and (2) IL-4/IL-5 synergistically recruit eosinophils through VCAM-VLA-4 adhesion pathways and bone marrow eosinopoiesis [114] (Figure 7). These Th2-polarized responses culminate in IgE-eosinophil axis activation, directly implicated in fungal-triggered allergic dermatitis pathogenesis [114,115]. This immunological dichotomy positions Th1/Th17-CD8^+^ circuits as antifungal executors, while Th2 deviation establishes a permissive microenvironment for chronic infection and allergic complications.

#### HI and dermatophytosis

While CMI orchestrates the primary defense against dermatophytes, emerging evidence reveals a nuanced role for HI, where select antibody classes exhibit adjuvant antifungal capacity under specific conditions. A prevailing hypothesis posits that robust antifungal antibody production enables HI-mediated protection, supported by clinical detection of pathogen-specific IgG and IgM in dermatophytosis patients – with *T. rubrum*-infected individuals showing elevated IgG levels and acute-stage IgM responses from serological analysis of patient samples [116] (Figure 7). Paradoxically, serum IgE levels rise in allergic dermatophytosis cases, yet these antibodies lack direct antifungal activity, instead correlating with mild inflammatory lesions in chronic recurrent infections from clinical IgE measurement studies [117]. Highly inflammatory presentations involve DTH, where IgM-allergen immune complexes activate complement cascades and recruit T cells to infection sites, bridging humoral and cellular immunity [118] (Figure 7).

Mast cell-mediated pathology emerges in *T. mentagrophytes* infections as fungal antigens crosslink surface-bound IgE, triggering histamine/proinflammatory mediator release [119] (Figure 7). This immunological duality mirrors observations in *Candida* infections, where HI generates both antifungal and nonprotective antibodies, with outcome determined by antibody specificity, pathogen-neutralizing potency (exemplified by IgG/IgM), and quantitative thresholds – factors critically influencing immune activation efficacy [120]. Thus, while CMI remains central, HI’s role in dermatophyte defense hinges on antibody class balance, functional specificity, and their interplay with inflammatory cascades.

### Critical appraisal of host defense studies

Despite the comprehensive characterization of immune responses summarized above, several systematic limitations warrant consideration:

**(1) Model limitations**: Most mechanistic insights derive from*in vitro*co-culture systems (keratinocytes or immune cells with fungal elements), which do not recapitulate the three-dimensional architecture of the skin or the temporal dynamics of infection. Animal models of dermatophytosis are challenging to establish due to species-specific differences in susceptibility, and most studies use immunosuppressed animals to achieve sustained infection, limiting translational relevance to immunocompetent hosts.

(2) **Evidence hierarchy:** The evidence base varies substantially across immune components. AMP and PRR studies are largely descriptive (expression profiling) with limited functional validation (knockout/knockdown). Cytokine and chemokine responses are well documented *in vitro*, but their *in vivo* relevance remains to be fully elucidated. CMI studies have the strongest clinical correlation (DTH status correlating with recovery vs chronicity), while HI studies remain largely correlative with unclear causality.
(3) **Contradictory findings:** Conflicting results exist regarding the protective vs pathogenic roles of certain immune responses. For example, while Th1/Th17 responses are generally considered protective, excessive inflammation can cause tissue damage. Similarly, whether antifungal antibodies are protective or merely disease markers remains debated.
(4) **Gaps in knowledge:** The relative contribution of each immune cell type to fungal clearance vs inflammatory pathology remains poorly quantified in human infections. The functional significance of HI in dermatophyte defense is still unresolved. Longitudinal studies tracking immune responses from initial infection through resolution or chronicity are lacking.
(5) **Clinical translation:** Despite extensive characterization of immune mechanisms, immunomodulatory therapies for dermatophytosis (beyond conventional antifungals) have not been developed. The potential for targeting specific immune pathways (e.g. enhancing Th1 responses, suppressing Th2 polarization) remains unexplored.

## Noncoding RNAs and dermatophytosis

NcRNAs, which are transcribed from over 90% of the human genome, represent a functionally diverse class of RNA molecules. Despite their non-coding nature, they critically orchestrate cellular processes – from transcriptional regulation and post-transcriptional modification to protein activity modulation – thereby governing metabolism, proliferation, and developmental pathways. Emerging research increasingly implicate ncRNAs in dermatophytosis pathogenesis, with recent studies elucidating their regulatory roles in fungal-host interactions, immune evasion, and inflammatory cascades. This synthesis of evidence positions ncRNAs as pivotal molecular players in dermatophyte infections, offering novel mechanistic insights into disease progression and potential therapeutic targets.

The following four subsections examine the current evidence for each ncRNA class in dermatophytosis. A striking hierarchy of evidence is evident across these classes: miRNAs are the best-studied (multiple studies, some functional validation), lncRNAs and circRNAs are supported by descriptive profiling studies (limited functional validation), and tRNAs/tRFs are at the exploratory stage (preliminary findings only). Summarized in the critical appraisals following each subsection, the field faces common challenges including lack of *in vivo* validation, absence of clinical correlates, and unresolved causality.

### MiRnas and dermatophytosis

#### Biogenesis and function of miRnas

**Figure 8.**
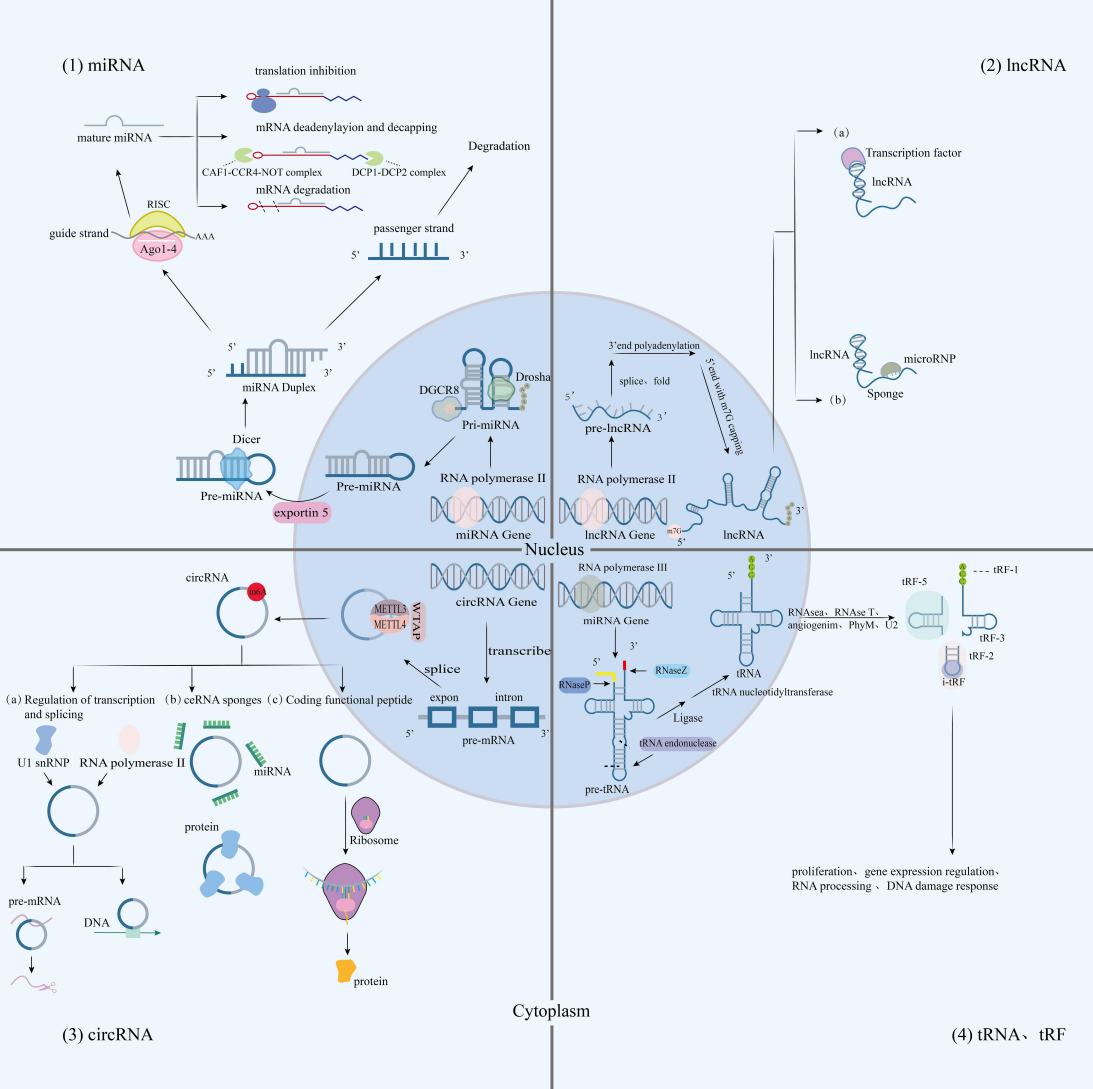
Transcription process of ncRNAs.

MiRNAs, a conserved class of 22-nucleotide endogenous ncRNAs first identified through the *lin-4* gene in *C. elegans* (which regulates larval development via RNA-mediated mechanisms rather than protein coding), constitute approximately 1% of the human genome while exerting widespread regulatory functions across species. Their biogenesis initiates with RNA polymerase II-mediated transcription of primary miRNA transcripts (pri-miRNAs) – long (hundreds to thousands of nucleotides), hairpin-structured ncRNAs featuring 5’ caps and 3’ poly-A tails [121] (Figure 8). Nuclear processing begins with the RNase III enzyme Drosha, which collaborates with DGCR8: Drosha binds the hairpin base while DGCR8 anchors the apical region, jointly cleaving pri-miRNAs to generate ~65-nucleotide precursor miRNAs (pre-miRNAs) [122] (Figure 8). These pre-miRNAs are then exported to the cytoplasm via Exportin-5 (Xpo5), where the RNase III enzyme Dicer recognizes their structural features through Platform-PAZ domain interactions, trimming them into transient miRNA duplexes [123] (Figure 8). Strand selection follows, with the passenger strand degraded and the guide strand incorporated into Argonaute (AGO) proteins via MID-PAZ domain protection, forming the RNA-induced silencing complex (RISC) to mediate post-transcriptional gene regulation [124] (Figure 8). This tightly coordinated maturation cascade underscores miRNAs as precision tools for eukaryotic gene expression control.

(1) miRNAs: Following transcription, processing, transport, and reprocessing, some miRNA genes generate mature miRNAs while others are degraded. Mature miRNAs, acting as guide strands, associate with AGO to form RISC, mediating post-transcriptional gene regulation. (2) lncRNAs: lncRNA gene transcripts undergo 5’m7G capping and 3’ polyadenylation in the nucleus before splicing and folding into mature lncRNAs, which regulate mRNA translation (a) and act as miRNA sponges (b). (3) circRNAs: circRNA genes are transcribed into pre-mRNA, which undergoes intronic splicing, back-splicing, and nucleotide modification to form mature circRNAs. These regulate cellular processes by modulating transcription/splicing via RNA polymerase II/U1 snRNP interactions (a), acting as ceRNA sponges for proteins and miRNAs (b), and encoding functional peptides through cap-independent translation (c). (4) tRNAs/tRFs: tRNA genes are transcribed and cleaved by RNase A, RNase T, angiogenins (RNY1 in yeast), PhyM, and U2 to generate tRNA-derived fragments (tRFs), which play crucial roles in proliferation, gene expression regulation, RNA processing, and DNA damage response.

#### miRnas in host immune responses to dermatophytes

**Figure 9.**
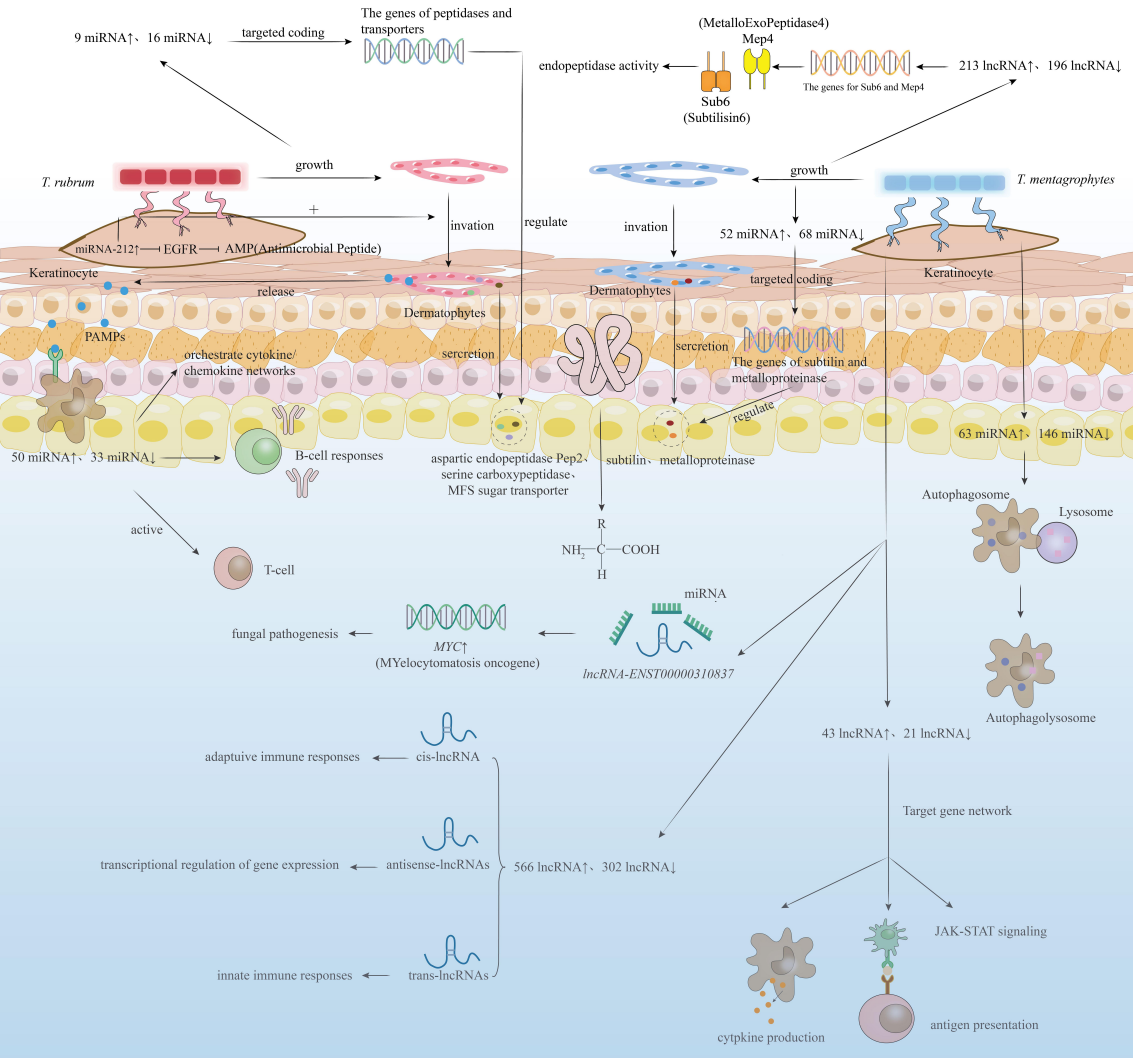
miRNAs, lncRNAs, and dermatophytosis.

MiRNAs critically regulate host-fungal interactions in dermatophytosis through multifaceted immunomodulatory mechanisms, as evidenced by pathogen-specific dysregulation patterns. In *in vitro T. rubrum*-infected keratinocyte models, upregulated miR-212 suppresses epidermal growth factor receptor (EGFR) signaling, impairing AMP production and facilitating fungal colonization [125] (Figure 9). Concurrently, using *in vitro* macrophage infection models, macrophages recognize *T. rubrum* PAMPs like mannans and adhesins via PRRs, initiating immune cascades that induce 50 miRNAs and suppress 33 others – these differentially expressed miRNAs orchestrate cytokine/chemokine networks, T-cell activation, and B-cell responses [126,127] (Figure 9). Parallel investigations using RNA-seq of *in vitro* infected keratinocytes in *T. mentagrophytes* models reveal profound miRNA reprogramming, with 63 upregulated and 146 downregulated species linked to autophagy regulation (macroautophagy, mitophagy) and fungal persistence mechanisms [128] (Figure 9). While these findings position miRNAs as central regulators of antifungal immunity and pathogenesis, the precise molecular interplay between specific miRNA candidates (e.g. miR-212), autophagy pathways, and fungal virulence factors requires systematic elucidation to translate mechanistic insights into therapeutic strategies.

During dermatophytosis, miRNAs orchestrate host-fungal interactions through immunomodulation and developmental control. In *T. rubrum*, miR-212 impairs EGFR signaling and AMP production, while PRR recognition triggers miRNA cascades that reshape cytokine/chemokine networks and T-cell responses. In *T. mentagrophytes*, miRNA reprogramming regulates autophagy and fungal persistence, with stage-specific miRNAs targeting peptidases (e.g. Pep2, subtilisins) and transporters to facilitate keratin degradation and hyphal invasion. Simultaneously, lncRNAs modulate host immunity via JAK-STAT signaling, antigen presentation, and innate/adaptive regulation, while enhancing fungal virulence through ceRNA mechanisms (e.g. lncRNA-ENST00000310837 elevating *MYC*) and stage-specific control of endopeptidases (e.g. Mep4, Sub6), collectively bridging fungal enzymatic activation with host immunometabolic responses.

#### miRnas regulating fungal virulence and development

Dermatophytes like *T. rubrum* and *T. mentagrophytes* transition from noninvasive conidia to an invasive mycelial phase, enabling tissue penetration and exacerbating infection as observed in *in vitro* culture models [129,130]. Emerging studies reveal miRNA-mediated regulation of fungal pathogenicity through developmental control: *in vitro* cultured *T. rubrum* at different developmental stages, 25 miRNAs (16 downregulated, 9 upregulated) modulate virulence by targeting genes encoding peptidases (aspartic endopeptidase Pep2, serine carboxypeptidase) and transporters (MFS sugar transporter) – key enzymes facilitating keratin degradation and nutrient acquisition during mycelial invasion [131] (Figure 9). Similarly, in *T. mentagrophytes in vitro* cultures, 119 stage-specific miRNAs (56 upregulated, 63 downregulated in hyphae) are enriched in endopeptidase activity, a critical virulence mechanism where subtilisins and metalloproteinases degrade stratum corneum proteins into assimilable peptides to establish infection [130] (Figure 9). These findings posit miRNAs as pivotal regulators of dermatophyte pathogenicity, potentially fine-tuning protease secretion and hyphal morphogenesis to optimize host colonization, though mechanistic validation of miRNA-target networks in fungal development remains an open frontier.

#### Critical appraisal

The studies summarized in this section derive predominantly from *in vitro* models using keratinocyte cell lines or isolated macrophages. Only a limited number of studies have employed *ex vivo* human skin explants, and no *in vivo* animal studies have been reported to date for miRNA regulation in dermatophytosis. Regarding species consistency, the miRNA signatures reported for *T. rubrum* and *T. mentagrophytes* show limited overlap, with miR-212 being specifically implicated in *T. rubrum* infections, whereas broader miRNA reprogramming (63 up, 146 down) is reported for *T. mentagrophytes*. Whether these differences reflect genuine species-specific regulatory mechanisms or arise from methodological variations (e.g. different RNA-seq platforms, infection time points, or fungal strains) remains unclear. Key gaps include: (1) the absence of functional validation via miRNA gain-of-function/loss-of-function approaches in relevant infection models, (2) lack of clinical studies correlating miRNA signatures with disease severity or treatment response in dermatophytosis patients, and (3) unresolved causality – whether observed miRNA changes drive pathogenesis or merely represent epiphenomena of infection. Future studies utilizing *in vivo* animal infection models, clinical cohorts, and CRISPR-based miRNA editing are warranted to address these limitations.

### LncRnas and dermatophytosis

#### Biogenesis and functional versatility of lncRnas

LncRNAs, defined as non-protein-coding transcripts exceeding 200 nucleotides without functional open reading frames, are primarily transcribed by RNA polymerase II and processed through mRNA-like maturation pathways, including 5’ m7G capping, splicing, and 3’ polyadenylation – before being retained in the nucleus or exported to the cytoplasm via nuclear pore complexes [124] (Figure 8). Functionally versatile, cytoplasmic lncRNAs regulate mRNA stability and translation, act as miRNA or protein sponges, and modulate organelle dynamics, while nuclear lncRNAs employ distinct biogenesis mechanisms exemplified by NEAT1 and MALAT1: these long, non-polyadenylated transcripts acquire triple-helical 3’ termini through RNase P processing, enabling nuclear retention and roles in chromatin remodeling, RNA polymerase I activity, nucleolar organization, and molecular decoy functions [124,132] (Figure 8). This structural dichotomy underpins lncRNA’s capacity to orchestrate gene expression across cellular compartments through context-dependent molecular interactions.

#### LncRnas in host immune modulation

LncRNAs, implicated in 168 human diseases spanning cancer (39.8%), cardiovascular (10.8%), and neurodegenerative disorders (8.4%), along with Crohn’s disease, diabetes, and muscular dystrophy, are emerging as key regulators in dermatophytosis pathogenesis [133]. In *T. mentagrophytes-*infected skin (*in vivo*), 64 differentially expressed lncRNAs (43 upregulated, 21 downregulated) correlate with critical immune pathways, including antigen presentation, JAK-STAT signaling, and cytokine production – via predicted target gene networks [134] (Figure 9). In *in vitro* infected keratinocyte models, KCs exposed to *T. mentagrophytes* exhibit 868 dysregulated lncRNAs (566 upregulated, 302 downregulated), classified functionally as *antisense*-lncRNAs (transcriptional regulation), *cis*-lncRNAs (adaptive immunity initiation), and *trans*-lncRNAs (innate immunity modulation) [135] (Figure 9). The competitive endogenous RNA (ceRNA) mechanism is exemplified by lncRNA-ENST00000310837, which sponges miRNAs to upregulate *MYC* expression, potentially driving fungal pathogenesis [128] (Figure 9).

#### LncRnas regulating fungal pathogenesis

RNA-seq analysis of *in vitro* cultured *T. mentagrophytes* at different developmental stages identified 409 stage-specific lncRNAs. These lncRNAs, which include *antisense*, *cis*, and *trans* subtypes, along with their associated ceRNA networks, were found to coordinate endopeptidase activity by targeting *Mep4* and *Sub6*. This finding underscores the dual role of lncRNAs in mediating both host immunity and fungal virulence [130] (Figure 9). This multilayered regulatory network positions lncRNAs as molecular bridges connecting fungal enzymatic activation (e.g. keratin-degrading endopeptidases) with host immunometabolic responses during dermatophyte invasion.

#### Critical appraisal

The emerging landscape of lncRNA regulation in dermatophytosis is based on descriptive profiling studies, with most data derived from bulk RNA-seq of infected keratinocytes *in vitro* without functional validation. While differential expression of lncRNAs (e.g. 868 dysregulated in *T. mentagrophytes*-infected KCs) has been robustly documented, mechanistic insights remain scarce. The proposed ceRNA mechanism for lncRNA-ENST00000310837 represents an interesting hypothesis, but direct experimental evidence – such as lncRNA-miRNA co-immunoprecipitation or luciferase reporter assays – is currently lacking. Across dermatophyte species, no comparative studies have been performed, leaving open the question of whether lncRNA responses are species-specific or conserved. Major controversies include the functional relevance of most dysregulated lncRNAs, given that the vast majority of lncRNAs exhibit low expression levels and poor sequence conservation. Furthermore, methodological challenges – such as the difficulty of distinguishing lncRNA-mediated effects from transcriptional noise – remain unaddressed in the current literature. To advance the field, future studies should prioritize: (1) functional validation using lncRNA knockdown/overexpression in relevant *in vitro* and *in vivo* models, (2) mechanistic dissection of lncRNA-protein and lncRNA-miRNA interactions, and (3) cross-species comparative analyses to identify conserved regulatory hubs.

### CircRnas and dermatophytosis

#### Biogenesis and functional mechanisms of circRnas

CircRNAs, characterized by covalently closed-loop structures, are predominantly formed through two biogenetic pathways: back-splicing – where a downstream 5’ splice site joins an upstream 3’ splice site of pre-mRNA to generate circular transcripts, and lariat-driven circularization, involving intron excision and stabilization of lariat structures into circRNAs [136]. Post-synthesis, circRNAs undergo modifications like N6-methyladenosine (m6A) before cytoplasmic export [137], and are classified into three subtypes based on sequence composition: exonic circRNAs, intronic circRNAs, and exon-intron circRNAs. Functionally versatile, circRNAs act as transcriptional modulators by interacting with RNA polymerase II or U1 snRNP, influence alternative splicing, serve as ceRNAs by sponging miRNAs (e.g. CDR1as/ciRS-7 targeting miR-7) or proteins (e.g. circ-FOXO3 sequestering senescence-associated proteins), and even encode functional peptides through cap-independent translation mechanisms (Figure 8). This structural and functional diversity positions circRNAs as dynamic regulators of eukaryotic gene expression.

#### CircRnas in host – pathogen interactions

Increasing evidence implicates circRNAs – now established as pivotal regulators across a spectrum of diseases including cancer, neuropsychiatry, metabolic disorders, and neurodegeneration – in dermatophytosis pathogenesis via stage‑specific expression patterns and dynamic host‑pathogen interactions. Using RNA-seq of *in vitro* infected keratinocytes, circRNAs exhibit stage-specific dysregulation during *T. mentagrophytes* infection, with 204 differentially expressed species (114 upregulated, 90 downregulated) identified in infected KCs – including validated candidates such as circ_002167, circ_0005035, novel_circ_005687, circ_0010131, and circ_0083444 showing marked upregulation, while circ_0081207 demonstrates significant downregulation [128] (Figure 9). Mechanistically, circ_0081207 is predicted to function as a ceRNA by sequestering miRNAs to suppress *TP53* expression, thereby potentially facilitating *T. mentagrophytes*-driven dermatophytosis progression through p53 pathway modulation as inferred from bioinformatic target prediction [128] (Figure 9). This ceRNA-mediated regulatory axis highlights circRNAs as molecular switches balancing tumor suppressor responses and fungal pathogenicity in cutaneous infections.

#### CircRnas regulating dermatophyte development

Studies using RNA-seq of *in vitro* cultured *T. rubrum* at different developmental stages reveal 940 stage-specific circRNAs modulating virulence factors like subtilisin-like proteases and ATP-binding cassette transporters during hyphal development [138], while RNA-seq analysis of *in vitro* cultured *T. mentagrophytes*, hyphae and spores shows 26 differentially expressed circRNAs targeting secondary metabolite biosynthesis and serine/threonine kinase signaling to enhance pathogenicity [138](Figure 9). These findings collectively position circRNAs as bidirectional modulators, governing fungal virulence enzymes (alkaline phosphatase, aspartyl aminopeptidase) via ceRNA networks while rewiring host TP53/p53 pathways through similar mechanisms, suggesting circRNA-targeted strategies may disrupt critical nodes in both fungal invasion and host immune dysregulation.

#### Critical appraisal

CircRNA research in dermatophytosis is still in its nascent stages, with all current findings derived from RNA-seq studies *in vitro* without independent validation. The identification of 204 differentially expressed circRNAs in *T. mentagrophytes*-infected KCs and 940 stage-specific circRNAs in *T. rubrum* provides a valuable resource, but the functional relevance of these candidates remains largely unexplored. The proposed ceRNA mechanism for circ_0081207 is intriguing yet unvalidated; direct evidence for circRNA-miRNA binding (e.g. AGO2-RIP, biotin-labeled pull-down) and functional rescue experiments are urgently needed. Across dermatophyte species, no comparative data exist, and the extent to which circRNA responses are specific to *T. mentagrophytes* versus *T. rubrum* cannot be assessed from current evidence. Key gaps include: (1) lack of validation cohorts (all findings from single studies), (2) absence of functional studies (e.g. circRNA knockdown/overexpression in *in vitro* or *in vivo* models), (3) unclear biogenesis mechanisms of circRNAs in the context of fungal infection, and (4) no clinical correlates linking circRNA expression to disease outcomes. Given the inherent stability of circRNAs (resistant to exonucleases), they hold promise as biomarkers, but this potential remains purely speculative at present.

### tRnas, tRfs, and dermatophytosis

#### Biogenesis and classification of tRfs

tRNAs, constituting approximately 15% of cellular RNA, were traditionally viewed as adaptor molecules in protein synthesis, responsible for decoding mRNA and delivering amino acids to ribosomes. However, advances in molecular biology have revealed that tRNAs play far more complex roles in cellular physiology, with tRNA mutations or deletions now implicated in the pathogenesis of various human diseases including neurodegenerative disorders, breast cancer, and Charcot-Marie-Tooth disease, demonstrating their critical involvement in maintaining cellular homeostasis beyond their canonical role in translation.

Under cellular stress conditions, tRNAs undergo enzymatic cleavage by RNase A, RNase T, angiogenin (RNY1 in yeast), PhyM, and U2 to generate tRFs, a newly identified class of small ncRNAs with diverse regulatory functions (Figure 8). These tRFs play crucial roles in multiple cellular processes including proliferation, gene expression regulation, RNA processing, and DNA damage response [139] (Figure 8). Based on their origin from precursor or mature tRNA, tRFs are classified into five distinct categories: tRF-5, tRF-3, tRF-1, tRF-2, and tiRNA [140] (Figure 8). Exhibiting remarkable tissue-specific, disease-specific, and spatiotemporal-specific expression patterns, tRFs demonstrate significant potential as molecular biomarkers for clinical diagnosis. Current research has established their important regulatory functions in various pathological conditions, particularly in cancer and cardiovascular diseases [141], highlighting their broad therapeutic and diagnostic applications.

#### Differential expression of tRnas and tRfs in dermatophytosis

**Figure 10.**
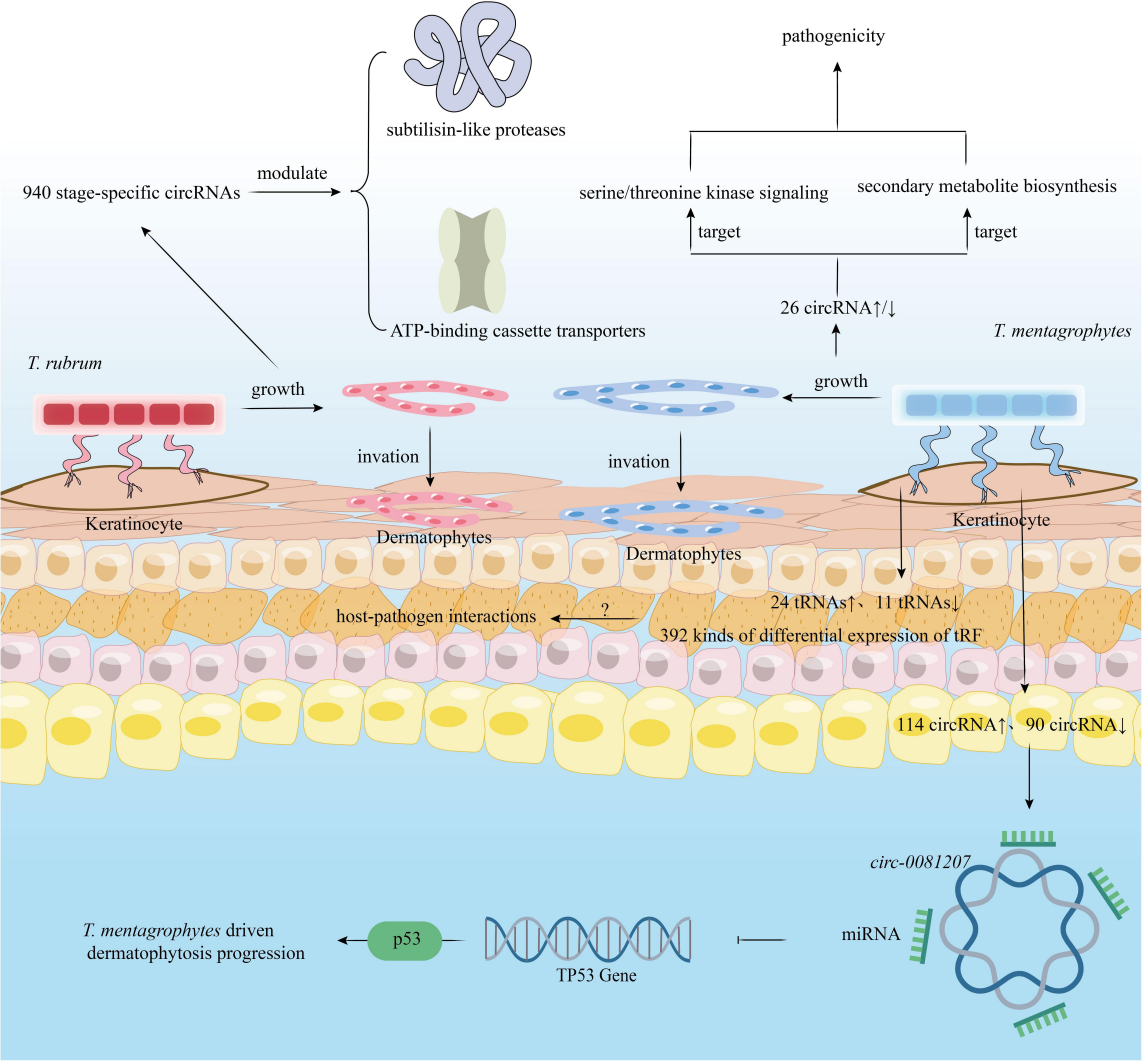
circRNAs, tRNAs, tRFs, and dermatophytosis.

Our preliminary findings from small RNA-seq of *in vitro* infected keratinocytes reveal that *T. mentagrophytes* infection induces significant changes in tRNA and tRF expression patterns in KCs, with 35 differentially expressed tRNAs identified (24 upregulated and 11 downregulated in infected vs non-infected cells) (Figure 10). Additionally, among 603 detected tRFs, 392 showed differential expression following fungal infection (Figure 10). While these pronounced alterations suggest a potential regulatory role for tRNA/tRF networks in host-pathogen interactions, the precise mechanisms linking these expression changes to dermatophytosis pathogenesis remain unclear (Figure 10). Further investigation of these dynamic expression patterns may provide crucial insights into the functional significance of tRNA/tRF modulation during fungal infection and open new avenues for understanding dermatophyte virulence mechanisms.

circRNAs in KCs post‑infection: 114 up‑regulated, 90 down‑regulated. circ‑0081207 may contribute to dermatophytosis by adsorbing miRNAs to inhibit p53 pathway. circRNAs in the fungus: 26 differentially expressed circRNAs in hyphae vs spores, potentially targeting metabolic synthesis to enhance pathogenicity. Stage‑specific circRNAs in *T. rubrum* hyphae: 940 circRNAs may regulate virulence factors and hyphal invasion. tRNAs/tRFs in KCs post‑infection: 24 tRNAs up‑regulated, 11 down‑regulated; 392 differentially expressed tRFs were detected, suggesting a regulatory role for the tRNA/tRF network in host – pathogen interactions.

#### Critical appraisal

The author’s preliminary findings represent the first evidence linking tRNA/tRF expression changes to dermatophytosis. These data derive from *in vitro* infected keratinocyte cultures and have not undergone peer review independently of this manuscript. As such, they should be interpreted with caution. While the identification of 35 differentially expressed tRNAs and 392 tRFs is descriptively informative, mechanistic insights are completely absent. The functional roles of specific tRFs – whether they act as signaling molecules, gene expression regulators, or stress response mediators – remain entirely unexplored in the context of fungal infections. Major gaps include: (1) no validation of the observed expression changes by orthogonal methods (e.g. qPCR, Northern blot), (2) no functional studies (e.g. tRF knockdown/overexpression in *in vitro* models), (3) no target identification (tRFs are known to regulate gene expression by associating with AGO proteins or by displacing translation factors, but none of these mechanisms have been investigated), and (4) no cross-species consistency data. The field of tRF biology in infectious diseases is rapidly evolving, and the findings reported here, while preliminary, may stimulate future investigations. However, at the current stage, conclusions should be limited to descriptive observations, and any functional claims would be premature.

## Conclusion

The pathogenesis of dermatophytosis is shaped by dynamic interactions between dermatophyte virulence and host resistance. Infection occurs when the pathogenicity of dermatophytes exceeds the host’s defensive capacity, whereas the host effectively eliminates the pathogen when its resistance surpasses fungal virulence. Dermatophytes primarily infect the host through adhesion, penetration, and colonization mechanisms mediated by secreted enzymes and cell wall proteins. Conversely, the host relies on both innate immunity (e.g. AMPs, PRRs, and chemokines) and adaptive immunity (cellular and humoral immune responses) to clear dermatophyte infections.

### Concluding remarks

Over the past decades, groundbreaking discoveries in ncRNA research have revolutionized our understanding of their roles in human diseases, including cancer, cardiovascular disorders, and neurodegenerative conditions. Small RNA-targeted therapeutics, exemplified by FDA/EMA-approved agents such as Nusinersen, Partisiran, Golodirsen, Givosiran, and Lumasiran, demonstrate the clinical translation potential of RNA-based interventions through their precisely characterized molecular mechanisms [142]. While the enigmatic functions of ncRNAs in fungal-host interactions are gradually being elucidated through *in vitro* and *ex vivo* studies [128], their specific regulatory mechanisms in the context of dermatophyte infections and host defense remain largely unexplored, particularly *in vivo*.

### Overall limitations of current research

Despite the significant advances summarized in this review, the field of dermatophytosis research faces several systematic challenges:

**(1) Model limitations**: The absence of robust, reproducible *in vivo* models that recapitulate human dermatophytosis in immunocompetent hosts remains a major bottleneck. Most animal studies rely on immunosuppression or mechanical barrier disruption, which may not reflect natural infection dynamics.

(2) **Translational gap:** While ncRNAs have emerged as promising therapeutic targets, delivery systems for cutaneous application (e.g. topical formulations of miRNA inhibitors or mimics) remain underdeveloped. The stratum corneum presents a formidable barrier for nucleic acid-based therapeutics.
(3) **Heterogeneity across studies:** Significant methodological heterogeneity (different fungal strains, infection doses, time points, RNA-seq platforms, and analytical pipelines) complicates cross-study comparisons and meta-analyses. Community-agreed standards for experimental design and data reporting are urgently needed.
(4) **Clinical validation deficit:** Most findings derive from *in vitro* or animal models; clinical cohorts with longitudinal sampling are rare. The prognostic or diagnostic value of candidate ncRNAs has not been validated in independent patient cohorts.
(5) **Fungal ncRNA biology:** Almost all ncRNA studies in dermatophytosis focus on host ncRNAs. The role of fungal-derived ncRNAs (e.g. fungal miRNAs or other small RNAs that may modulate host immunity) remains a completely unexplored frontier.

### Future directions and research priorities

Advances in next-generation sequencing, bioinformatics, and molecular biology techniques have enabled the systematic discovery of ncRNAs associated with dermatophytosis, elucidating their functional roles and mechanistic contributions to fungal pathogenesis using *in vitro* models [128,135,138]. To overcome current limitations in translating ncRNA research into clinical applications for dermatophytosis, future investigations should focus on key strategies to advance this field. A critical priority is expanding the catalog of dermatophyte-host interaction-associated ncRNAs. Cutting-edge technologies – such as long-read sequencing, single-cell RNA-seq, and spatial transcriptomics – now enable systematic ncRNA characterization, accelerating the discovery of novel ncRNA subtypes and their previously unrecognized regulatory roles [143]. The rapid evolution of bioinformatics tools unlocks transformative potential for developing skin-protective interventions targeting ncRNAs. Leveraging multi-omics datasets, including high-throughput transcriptomics, dermatophytosis model analyses, and integrative network medicine approaches enable systematic associations between ncRNA dynamics and clinical outcomes (e.g. disease risk, prognosis) [144]. Furthermore, these methodologies facilitate the identification of ncRNA-drug predictive pairs, offering a robust framework for discovering precision therapeutics with enhanced efficacy against dermatophytosis.

To advance ncRNA-based interventions, comprehensive functional characterization of dermatophyte-host interaction-linked ncRNAs is imperative, including identification of their upstream modulators (e.g. transcription factors, epigenetic regulators) and downstream effectors (e.g. immune signaling pathways, fungal virulence genes) [145]. Mechanistic insights must be validated through preclinical *in vivo* models (e.g. murine dermatophytosis) and clinical cohort analyses. While no ncRNA-targeted clinical trials for dermatophytosis are currently registered, emerging studies highlight their diagnostic and prognostic potential. Innovations in delivery systems (e.g. lipid nanoparticles, exosome-based carriers) now enable cell-specific ncRNA targeting [146]. Nevertheless, personalized therapeutic strategies leveraging ncRNAs to improve patient outcomes remain unexplored.

In summary, ncRNAs have emerged as pivotal regulators in dermatophytosis pathogenesis. Further translational studies integrating mechanistic investigations (e.g. gene editing, ncRNA-protein interaction mapping) and clinical validation (e.g. longitudinal cohort analyses) are anticipated to clarify their functional roles and therapeutic potential in this disease.

## Data Availability

Data sharing is not applicable to this article as no new data were created or analyzed in this study. This review is based entirely on previously published studies, all of which are cited in the reference list.

## Abrreviations

ATMT: Agrobacteriumtumefaciens-mediated transformation
*T.* *rubrum*: *Trichophyton rubrum*
*T.* *mentagrophytes*: *Trichophyton mentagrophytes*
*M.* *canis*: *Microsporum canis*
AMPs: Antimicrobial peptides
PRRs: Pattern recognition receptors
PAK: p21-activated kinase
NcRNAs: Non-coding RNAs
miRNAs: MicroRNAs
lncRNAs: Long non-coding RNAs
circRNAs: Circular RNAs
tRNA: Transfer RNA
tRFs: tRNA-derived fragments
KCs: Keratinocytes
DCs: Dendritic cells
PAMPs: Pathogen-associated molecular patterns
ROS: Reactive oxygen species
APCs: Antigen-presenting cells
MEP: Metalloprotease
Sub: Subtilisin
DTH: Delayed-type hypersensitivity
CDO: Cysteine dioxygenase
CS: Citrate synthase
IL: Isocitrate lyase
MS: Malate synthase
GPI: Glycosylphosphatidylinositol
